# Impact of low-intensity pulsed ultrasound on transcription and metabolite compositions in proliferation and functionalization of human adipose-derived mesenchymal stromal cells

**DOI:** 10.1038/s41598-020-69430-z

**Published:** 2020-08-13

**Authors:** Denggao Huang, Yuanhui Gao, Shunlan Wang, Wei Zhang, Hui Cao, Linlin Zheng, Yang Chen, Shufang Zhang, Jie Chen

**Affiliations:** 1 Department of Central Laboratory, Affiliated Haikou Hospital of Xiangya Medical College, Central South University, Haikou, 570208 Hainan China; 2 Department of Electrical and Computer Engineering, University of Alberta, Edmonton, AB T6G 2V4 Canada

**Keywords:** Biomedical engineering, Electrical and electronic engineering

## Abstract

To investigate the effect of low-intensity pulsed ultrasound (LIPUS) on the proliferation of human adipose-derived mesenchymal stromal cells (hASCs) and uncovered its stimulation mechanism. LIPUS at 30 mW/cm^2^ was applied for 5 min/day to promote the proliferation of hASCs. Flow cytometry was used to study the cell surface markers, cell cycle, and apoptosis of hASCs. The proliferation of hASCs was detected by cell counting kit-8, cell cycle assay, and RT-PCR. The expression of hASCs cytokines was determined by ELISA. The differences between transcriptional genes and metabolites were analyzed by transcript analysis and metabolomic profiling experiments. The number of cells increased after LIPUS stimulation, but there was no significant difference in cell surface markers. The results of flow cytometry, RT-PCR, and ELISA after LIPUS was administered showed that the G_1_ and S phases of the cell cycle were prolonged. The expression of cell proliferation related genes (*CyclinD1* and *c-myc*) and the paracrine function related gene (*SDF-1α*) were up-regulated. The expression of cytokines was increased, while the apoptosis rate was decreased. The results of transcriptome experiments showed that there were significant differences in 27 genes;15 genes were up-regulated, while 12 genes were down-regulated. The results of metabolomics experiments showed significant differences in 30 metabolites; 7 metabolites were up-regulated, and 23 metabolites were down-regulated. LIPUS at 30 mW/cm^2^ intensity can promote the proliferation of hASCs cells in an undifferentiating state, and the stem-cell property of hASCs was maintained. *CyclinD1* gene, *c-myc* gene, and various genes of transcription and products of metabolism play an essential role in cell proliferation. This study provides an important experimental and theoretical basis for the clinical application of LIPUS in promoting the proliferation of hASCs cells.

## Introduction

As research into our understanding of possible applications, stem cell research advances, stem cell therapies have been used to treat multiple diseases: angiogenesis^1^, diabetes^2^, hepatic failure^3^, multiple scleroses^3,4^, spinal cord injury^5^, glaucomas^6^, and Crohn’s diseases^7^. In treating these diseases, stem cell therapies have achieved promising results.

Mesenchymal stromal cells (MSCs) are separated from bone marrows, and they are a group of non-phagocytes with morphological characteristics of fibroblast-like adherent cells. Subsequent studies have shown that MSCs have been successfully separated from different organs, e.g., bone marrow derived mesenchymal stromal cells (BM-MSCs), adipose-derived mesenchymal stromal cells (ASCs), stem cells from the umbilical cord, soft bone and skin^8,9^. Because ASCs have abundant sources and can be easily obtained, they are becoming the primary source of seeding cells for next-generation tissue engineering^10^.

With the rapid development of stem cell research in recent years, the studies and applications of stem cell biology have become more advanced. hASCs have been widely used in diabetes, heart repair, Parkinson’s disease, bone healing, wound healing, and tumor treatments. Consequently, the success of hASC applications promotes the development of stem cell transplantation and regenerative medicine^10–12^. The maximum number of ASCs obtained from 100 mL adipose tissue is approximately 9.06 × 10^5^ stem cells^12^. However, the minimum number of stem cell transfusion therapy requires 2 × 10^6^ cells, while most treatment needs 10^8^ cells. A cell therapy course would require a substantial amount of ASCs cells^13^, which cannot be obtained from patients themselves or donators, and thus limits the application of hASCs. Therefore, the exploration and development of high-efficiency in vitro expansion of stem cells and the shortening of treatment time will be beneficial to patients in need of stem cells.

A cell’s microenvironment refers to the local physiological surroundings composed of cells, extracellular matrix, stromal cells, cytokines, and immune cells. Cell–cell interactions, cytokine interactions are expressed in this microenvironment. The morphology and biological behavior of cells, and even the differentiation of cells, are affected by the chemical composition of the extracellular matrix^14^. Stem cells, and their local microenvironment (or niche) regulate cell fate and behavior through signaling under the action of external mechanical forces. In vitro, synthetic models of stem cell niches can be used to precisely control and manipulate the biophysical and biochemical properties of the stem cell microenvironment to guide stem cell differentiation and function. The fundamental insights on the mechanisms of mechanics stem-cell biology also provide information for the design of artificial cells to support stem cell regeneration therapy^15,16^.

Low-intensity pulsed ultrasound (LIPUS) transmits mechanical stimulation through the skin to biological tissues in the form of high frequency, small amplitude, and pulsed pressure waves. LIPUS, as a non-invasive treatment method, has been widely applied^17–19^. Since Duarte and Xavier first reported the success of LIPUS in treating refractory bone nonunion in 1983; successive generations of LIPUS devices have been developed. Previous studies have shown that LIPUS could stimulate the synthesis of DNA and proteins in vascular cells^20–22^, as well as promote cell activity, cytokine release, gene expression, etc.^23–25^, indicating that LIPUS can be used in clinical applications. Some researchers are using LIPUS to stimulate the proliferation of human hematopoietic progenitor stem cells (hHSCs)^26^, h-BM-MSCs^27,28^, human amniotic mesenchymal nerve^29^, and neural crest stem cells derived from pluripotent stem cells^30^. These results suggest that more numbers of stem cells can be obtained through LIPUS stimulations for clinical application. However, the intensity and stimulation duration of LIPUS acting on different cells were inconsistent, and the changes in the biochemical and metabolic components in the cells after the stimulation and the specific mechanism were still unclear, which deserves further study.

Researchers have made significant progress in using LIPUS to stimulate other types of stem cells. However, the effect of LIPUS stimulation on the proliferation and function of ASCs has rarely been reported, and its mechanism was remain unclear. Here, our study showed that 30 mW/cm^2^ of LIPUS could promote the effective proliferation of hASCs. Cell proliferation may be the result of the up-regulation of *Cyclin D1* and *c-myc* genes as well as the regulation of transcriptional genes and metabolites through a variety of pathways. These results may provide substantial evidence supporting the use of LIPUS in promoting stem cell activity and proliferation. Tissue engineering and clinical therapy may benefit from the use of LIPUS proliferated stem cells.

## Materials and methods

### Reagents

hASCs and its culture media components were purchased from Guangzhou Cyagen Biosciences co., LTD (Guangzhou, China). Penicillin–streptomycin, fetal bovine serum (FBS), Dulbecco's Phosphate Buffered Saline (D-PBS), and Trypsin were purchased from Gibco (USA). CD105-PE, CD73-PE, HLA-ABC-PE, CD14-FITC, CD34-PE, and CD45-PE antibody were purchased from Becton–Dickinson (USA). Trizol reagent was purchased from Life Technologies (USA). MiniBEST Universal RNA Extraction Kit, SYBR Premix Ex Taq II (Tli RNaseH Plus) kit, and PrimeScript RT Master Mix kit (Perfect Real Time) were purchased from TaKaRa (Japan). RNeasy Mini Kit was purchased from Qiagen (Germany). Cell cycle kit and Annexin V-FITC/PI apoptosis kit were purchased from Multisciences (Lianke) Biotech, co., LTD (Hangzhou, China). Cell Counting Kit-8 (CCK-8) kits were purchased from Dojindo (Osaka, Japan). Ki-67 cell proliferation kit (IF) was purchased from Sangon Biotech (Shanghai) Co., Ltd (Shanghai, China). Enzyme-Linked Immunosorbent assay kit was purchased from Abcam (United Kingdom). The Scepter 2.0 cell counter was purchased from Merck Millipore (Massachusetts, USA). The chromatographic column was purchased from Waters (USA). Acetonitrile and methanol were purchased from Merck (Darmstadt, Germany). Formic acid was purchased from CNW Technologies (Shanghai, China).

### Ultrasound stimulation device

SonaCell (IntelligentNano Inc. Canada) is used to generate LIPUS at 1.5 MHz, with pulse repetition of 1 kHz at a 20% duty cycle. Average output intensity adjusted between 0 mW/cm^2^ to 80 mW/cm^2^. The ultrasound transducer was attached to the bottom of the cell culture dish. Ultrasound gel was applied to help the transmission wave of ultrasound entering the cells. In this study, LIPUS intensities of 10 mW/cm^2^, 20 mW/cm^2^, 30 mW/cm^2^, 40 mW/cm^2^, 50 mW/cm^2^, 60 mW/cm^2^, 80 mW/cm^2^ were used for cell stimulation in the stimulated experimental group while 0 mW/cm^2^ was used as the control group. To avoid LIPUS wave interference, only 6 holes were used in the 12-hole plate.

### Cell culture and ultrasound stimulation

The passage 3(P3), passage 6(P6), and passage 8(P8) of hASCs were collected. The cell density was adjusted to 2 × 10^4^ cells /mL, and 1 mL cell suspension was inoculated into the wells marked with corresponding labels in the 12-well plate. After 24 h of cell culture, the medium was refreshed and completely replaced, and hASCs were stimulated by LIPUS. During the stimulation, the device and the cells were both placed in the incubator, Subject to stimulation durations of 5 min/dose, 4 times of continuous stimulation at 24 h, 48 h, 72 h and 96 h of cell culture, respectively. After stimulation, the cells were cultured in the incubator (37 °C, 5% CO_2_) for 24 h, and the cells were collected at their respective time point and analyzed.

### CCK-8 assay

When the stimulated cells were cultured to the detection time point, the medium was removed, and then 200μL of fresh medium was added. The CCK-8 solution of 20μL was added to each well and placed in the incubator for 4 h. Then 100 μL of supernatant was transferred to a 96-well plate, and OD value at 450 nm wavelength was detected by the iMark plate reader.

### Flow cytometry assay

The cells stimulated by LIPUS were digested with 0.25% trypsin–EDTA and collected. The expressions of hASCs surface markers CD105-PE, CD73-PE, HLA-ABC-PE, CD14-FITC, CD34-PE, and CD45-PE were determined by flow cytometry. The cells were removed and washed with D-PBS buffer twice. 3 × 10^5^ /mL of hASCs were resuspended in the D-PBS, and then centrifuged at 3,000 rpm for 5 min; after the supernatant was removed, the fluorescent monoclonal antibody was added and incubated at room temperature in the dark for 30 min and detected in the flow cytometry machine (Becton–Dickinson, USA).

### Enzyme linked immunosorbent assay (ELISA)

The supernatant of the cell culture medium in the stimulation group and the control group were collected, centrifuged at 3,000 rpm for 20 min, and then the supernatant was collected. According to the manufacturer's instructions for the kit, the concentration of EGF, FGF-2, IL-6, and IL-2 in the samples were detected by the iMark plate reader.

### RT-PCR assay

Trizol reagent and the RNA Extraction column purification Kit MiniBEST Universal RNA Extraction Kit were used to extract total RNA from hASCs cells in the stimulation group and the control group. Referring to the manufacturer's instructions of PrimeScript RT Master Mix kit, the reverse transcriptional reactions reagents were prepared. After gentle mixing, the reverse transcription reaction is carried out to synthesize the first strand of cDNA. The fluorescence quantitative PCR reaction system was prepared according to the manufacturer's instruction manual of SYBR Premix Ex Taq II (Tli RNaseH Plus) kit. After fully mixing, it was placed in a real-time fluorescence PCR instrument for fluorescence quantitative PCR reaction. Experimental conditions: pre-denaturation 95 ℃, 30 s; PCR reaction (50 cycles): 95 ℃, 5 s; 55 ℃, 30 s; 72 ℃, 30 s. The relative expression levels of different genes were normalized to the GAPDH gene. The primer is shown in Table 1.

**Table 1:** Primers for RT-PCR.

Primer	Primer sequence
GAPDH-f	ATCACCATCTTCCAGGAGCGA
GAPDH-r	TTCTCCATGGTGGTGAAGACG
VEGF-f	CCCACTGAGTCCAACAT
VEGF-r	TTTCTTGCGCTTTCGTTTTT
TB4-f	TGCTTGCTTCTCCTGTTCAA
TB4-r	ACAAACCCGATATGGCTGAGATCGAG
SDF-1α -f	CTACTCTCTCCCCGACTCCG
SDF-1α -r	AAGCAGGGGGACCATTACAC
MCP-1-f	CCCCAGTCACCTGCTGTTAT
MCP-1-r	TGGAATCCTGAACCCACTTC
COMP-f	AGGGAGATCGTGCAGACAA
COMP-r	AGCTGGAGCTGTCCTGGTAG
RUNX2-f	TCTTCACAAATCCTCCCC
RUNX2-r	TGGATTAAAAGGACTTGGTG
OCN-f	TACCTGTATCAATGGCTGG
OCN-r	GAGTTTATTTGGGAGCAGCT
Sox9-f	CGCCATCTTCAAGGCGCTGC
Sox9-r	CCTGGGATTGCCCCGAGTGC
BMI-1-f	CTGGTTGCCCATTGACAGC
BMI-1-r	CAGAAAATGAATGCGAGCCA
cyclinD1-f	AATGACCCCGCACGATTTC
cyclinD1-r	TCAGGTTCAGGCCTTGCAC
c-myc-f	AAACACAAACTTGAACAGCTAC
c-myc-r	ATTTGAGGCAGTTTACATTATGG
ACAN-f	CCAGTGCACAGAGGGGTTTG
ACAN-r	TCCGAGGGTGCCGTGAG
LRP5-f	ACCGGAACCACGTCACAG
LRP5-r	GGGTGGATAGGGGTCTGAGT
ALPL-f	GGACCATTCCCACGTCTTCA
ALPL-r	CAGGCCCATTGCCATACA
COL2A1-f	CCAGTTGGGAGTAATGCAAGGA
COL2A1-r	ACACCAGGTTCACCAGGTTCA

### Apoptosis assay

The stimulated hASCs were digested with trypsin, counted by cell counter, and 5 × 10^5^ cells were collected. Then Annexin V-FITC and propidium iodide (PI) were added. Annexin V-FITC (Ex = 488 nm, Em = 530 nm) was detected by the FITC channel (FL1), and PI was detected by the PE detection channel (FL2) on the flow cytometry machine.

### Cell cycle analysis

hASCs from the stimulation group and the control group were collected and labeled accordingly. The cell cycle kit (Multisciences (Lianke) Biotech, co., LTD) was analyzed according to the manufacturer's instructions. The cells were collected and adjusted to 1×10^6^ cells/mL. Ethanol (v/v, 70%) was added for fixation, and PI was used to stain the nucleus. The red fluorescence at 488 nm was recorded.

### Transcript analysis

The standard operating procedure of RNeasy Mini Kit was used for total RNA extraction, then mRNA enrichment and rRNA removal, fragmentation, synthesis of one- and two-strand cDNA, end-filling, 3′-end addition A, splicing, PCR amplification, library quality control, library standardization and clustering, and sequencing. The original sequencing data was processed by Fastp software through the disjointing sequence and low-quality sequence. The data after quality control were compared to the Homo sapiens genome (hg38); the HISAT2 v.2.0.5 software was used. The matched reads were further annotated and quantified with stringtie software (Johns Hopkins University, USA), and the R package edagR (Justus-Liebig- Universitat, Germany) was used for standardization and statistics analysis.

### Metabolomic profiling

Sample pretreatment: 1,000 μL methanol:acetonitrile : water (V/V, 4:4:2) solution was added to the tube containing cells, vortexed for 60 s, and ultrasonic crushing was conducted in a water bath at 4℃ for 10 min. The solution was placed in liquid nitrogen for 1 min, warmed to room temperature, and immersed in a water bath at 4℃ for ultrasonic crushing for 10 min (repeated 3 times). The samples were placed at − 20℃ for 1 h and centrifuged at 4℃ with 13,000 rpm for 15 min and then the supernatant was extracted for testing. Untargeted metabolomics analysis was performed by LC/MS Data Acquisition (Version B.08.00, Agilent Technologies, USA) coupled with QTOF 6,545 (Agilent Technologies, USA). For chromatographic separation, ACQUITY UPLC HSS T3 column (2.1 mm × 100 mm, 1.8 μm, Waters, USA) was used at 35 °C. Parameters of mass spectrometry were as follows: the gas temperature was 320 °C and gas flow of 8 L/min, sheath gas flow of 12 L/min, and sheath gas temperature maintained at 350 °C. Capillary voltage (VCap) was the anion mode of 3,500 V and the positive ion mode of 4,000 V.

Data Processing and Analysis: MSDIAL software was used to perform peak search, peak alignment, normalization, and other data processing on the data. Meanwhile, metlin, MassBank, MoNA, and HMDB databases were independently integrated based on the first and second level database search, and the identification results were obtained. For the identified data of MSDIAL alignment, the coefficient of variation (CV) value of the sample index was controlled by quality control (QC) samples to be less than 50%, and the ion peak with the missing value greater than 50% in the group was deleted. The auto-scaling method was applied for normalization, and MetaboAnalyst 4.0 software was applied for difference analysis and enrichment analysis. The quality error of the first level is controlled at 10PPM. The retention time limit is less than 0.15 min. After alignment, the unidentified difference indicators are re-identified to improve the identification rate of the different indicators.

### Statistical analysis

For all measurements, we have performed at least three independent experiments. All data was collected and analyzed using SPS 20.0 statistical software. Means ± standard deviation (SD) were used to present quantitative data. The two independent samples t-test was used to compare two group data, and the one way-ANOVA method was used to compare more group data. *p* < 0.05 was considered statistically significant.

## Results

### Selection of optimal LIPUS stimulation conditions

**Figure 1:**
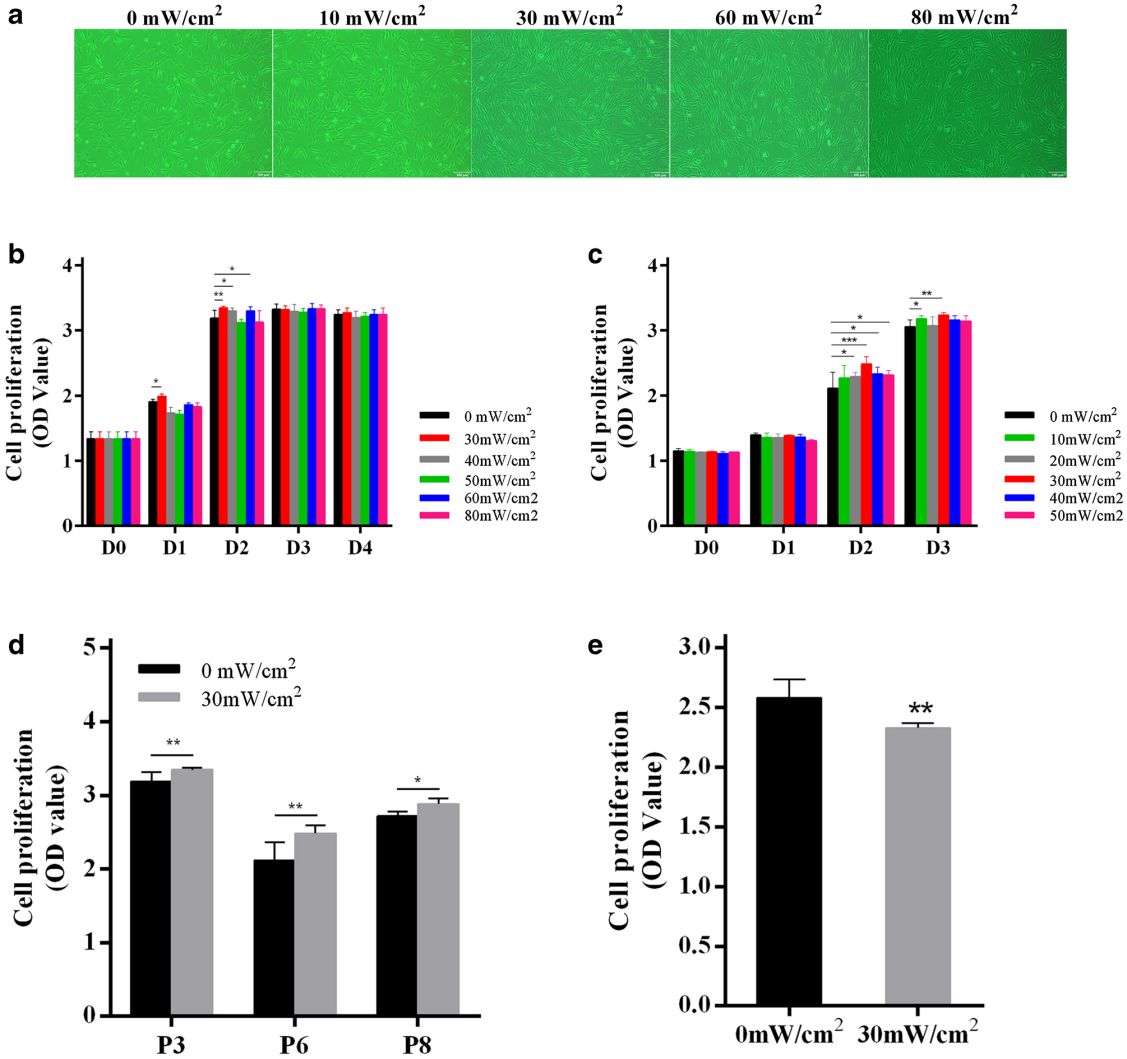
The results of hASCs cell morphology and CCK-8 assay with or without LIPUS stimulation. (**a**) The cell morphology of hASCs. Scale bar, 100 µm. (**b**) The cell viability of hASCs without or with 30 mW/cm^2^, 40 mW/cm^2^, 50 mW/cm^2^, 60mW/cm^2^, and 80mW/cm^2^ intensities of LIPUS, respectively. (**c**) The cell viability of hASCs without or with 10 mW/cm^2^, 20 mW/cm^2^, 30 mW/cm^2^, 40 mW/cm^2^, and 50 mW/cm^2^ of intensities of LIPUS, respectively. (**d**) The cell proliferation of different cell passages. P3, P6, and P8 are the abbreviation of Passage 3, Passage 6, and Passage 8, respectively. (**e**) The cell proliferation of P3 hASCs after a 10-min simulation per day. Error bars represent the SD of quadruplicate samples in (**b**) and (**c**), and of triplicate samples in (**d**) and (**e**). *p < 0.05, **p < 0.01, ***p < 0.001.

To find the optimal intensity for LIPUS to stimulate the growth of hASCs, the LIPUS generating device, SonaCell, was set to intensities between 30–80 mW/cm^2^. The stimulation lasted 5 min for each instance for 4 consecutive days. After 72 h, the cell viability of 30 mW/cm^2^, 40 mW/cm^2^, and 60 mW/cm^2^ of LIPUS was better than the control (*p* < 0.05) as discovered by the CCK-8 kit. Among the groups, 30 mW/cm^2^ was the best with a significant difference (Fig. 1b). Then, LIPUS intensities were further reduced to between 10 and 50 mW/cm^2^. The results showed that 20 mW/cm^2^, 30 mW/cm^2^ and 40 mW/cm^2^ of LIPUS can stimulate hASCs proliferation, with 30 mW/cm^2^ intensity achieving the best results (Fig. 1c). A similar treatment for the hASCs of P3, P6, and P8 was studied. All results showed that 30 mW/cm^2^ of LIPUS had the best positive effects on hASCs (Fig. 1d). To uncover the impact of stimulation duration on the proliferation of hASCs, 10 min of LIPUS was administered. The results showed that the cell viability experienced an obvious decrease compared to the control (*p* < 0.01) (Fig. 1e). Therefore, the 30 mW/cm^2^ intensity of LIPUS and 5 min/day treatment was selected for our late experiments (Supplementary Fig. 1). The change of fibroblast-like cell morphology was not observed in with and without LIPUS stimulations by microscope (Fig. 1a).

### Analysis of hASCs cell surface markers after stimulation by LIPUS

To investigate whether the cell surface markers of hASCs were changed after LIPUS stimulation, the surface markers of adipose mesenchymal stromal cells (CD73, CD105, HLA-ABC, CD45, CD34, and CD14) were detected by flow cytometry. The results showed that there was no significant difference in cell surface markers in the stimulation group (30 mW/cm^2^) compared with that of the control group (0 mW/cm^2^) (Table 2, Supplementary table, and Supplementary Fig. 2).

**Table 2:** Comparison of hASC cell surface markers between the stimulation group and the control group (P3, n = 3).

	CD73	CD105	HLA-ABC	CD45	CD34	CD14
0 mW/cm^2^	99.02 ± 1.02	99.63 ± 0.55	81.23 ± 1.45	3.80 ± 0.79	7.46 ± 0.49	0.33 ± 0.15
30 mW/cm^2^	99.33 ± 0.65	99.53 ± 0.64	84.60 ± 2.02	2.73 ± 2.02	7.86 ± 0.15	0.50 ± 0.17

The expressions of hASCs surface markers (CD105-PE, CD73-PE, HLA-ABC-PE, CD14-FITC, CD34-PE, and CD45-PE) were determined by flow cytometry in the stimulation group and the control group. The experimental data were statistically analyzed using two-independent sample t-tests. Means ± standard deviation (SD) were used to present quantitative data.

**Figure 2:**
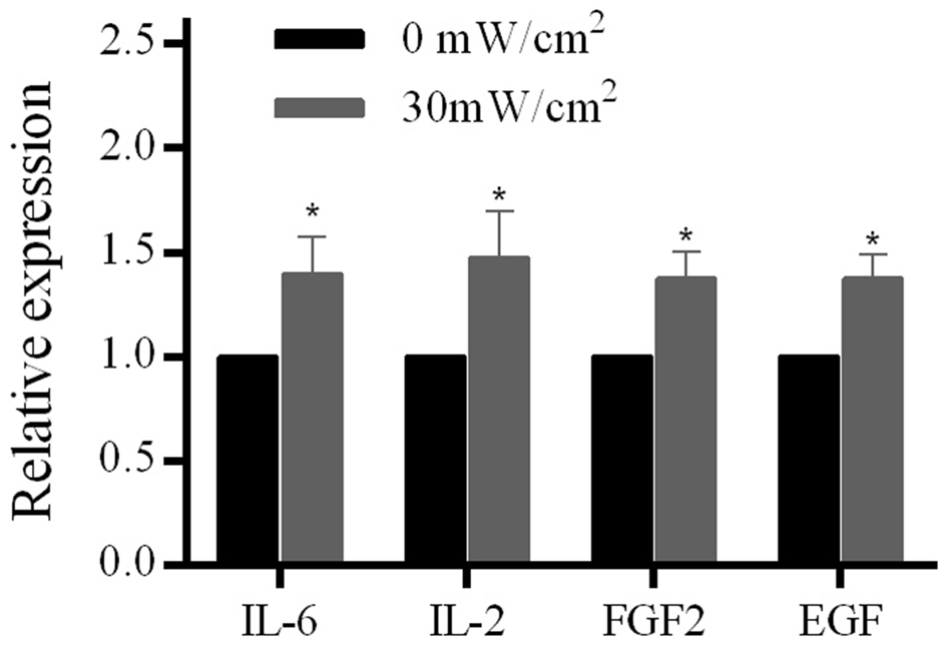
The cytokines expression of hASCs with and without LIPUS stimulation. The concentrations of EGF, FGF-2, IL-6, and IL-2 in the supernatant of cell culture medium were measured by ELISA and detected in iMark plate reader.

### Results of cytokine detection

To understand the cytokine secretion potential of hASCs after stimulation by LIPUS, the cytokines of IL-6, IL-2, FGF2, and EGF were measured by ELISA. The results showed that the relative expressions of IL-6, IL-2, EGF, and FGF-2 in the supernatant of hASCs cell culture medium in the stimulation group were up-regulated compared with that of the control group (*p* < 0.05) (Fig. 2).

### Results of apoptosis assay

**Figure 3:**
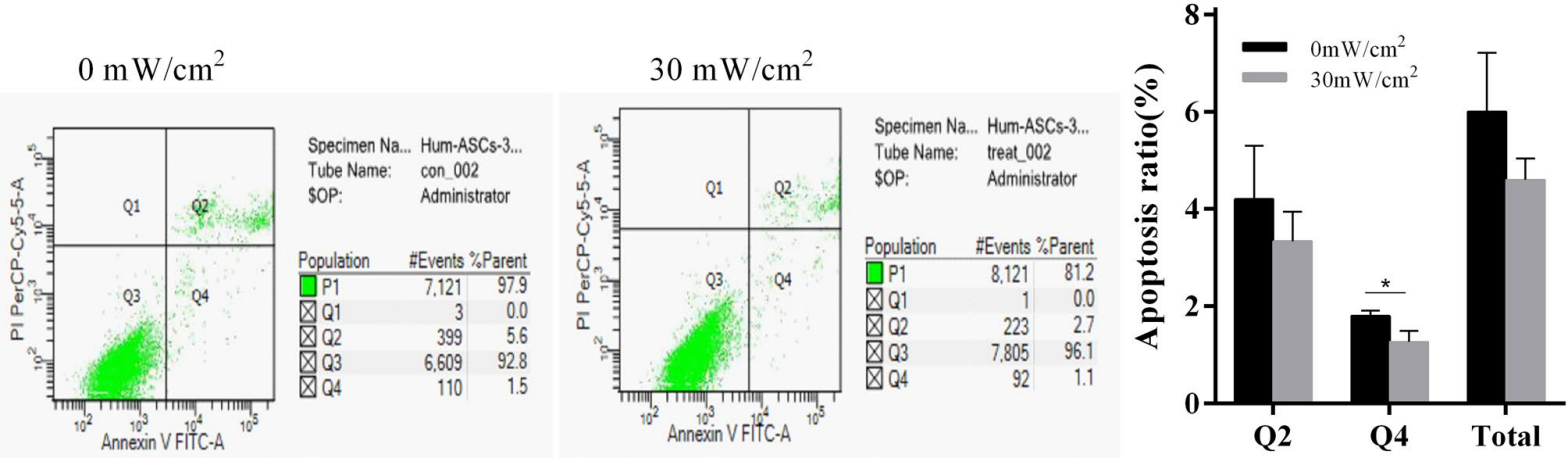
The analysis of hASCs apoptosis in the stimulation group and control group. The two groups of hASCs were stained by Annexin V-FITC and PI. Annexin V-FITC (Ex = 488 nm, Em = 530 nm) was detected by the FITC channel (FL1), and PI was detected by the PE detection channel (FL2) on flow cytometry machine.

To analyze the apoptosis of hASCs after treatment by LIPUS, Annexin V-FITC/PI apoptosis kit was used in detecting hASCs apoptosis. The results showed that there was no difference in late apoptosis (Q2) between the two groups, while the early apoptosis (Q4) in the stimulation group was decreased (*p* < 0.05) (Fig. 3).

### Results of cell cycle analysis

**Figure 4:**
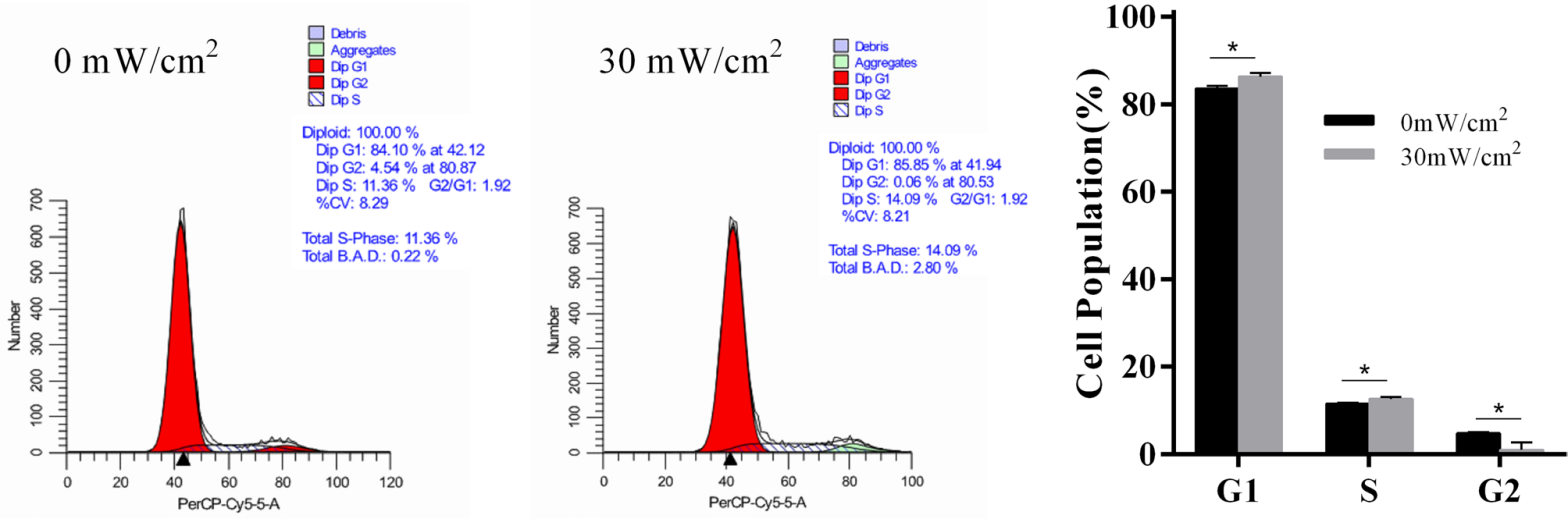
The cell cycle distribution of hASCs with and without LIPUS stimulation. The cells were collected and adjusted to 1 × 10^6^ cells /mL. Ethanol (v/v, 70%) was added for fixation, and propidium iodide (PI) was used to stain the nucleus. The red fluorescence at 488 nm was recorded.

To analyze the cell cycle of hASCs treated by LIPUS, flow cytometry was used to detect the cell cycle of hASCs. The results showed that the G1 phase and S phase of cells in the stimulation group were higher than those in the control group (*p* < 0.05). The G2 phase was significantly lower in the stimulation group than those in the control group. (*p* < 0.05) (Fig. 4).

### Results of RT-PCR assay

**Figure 5:**
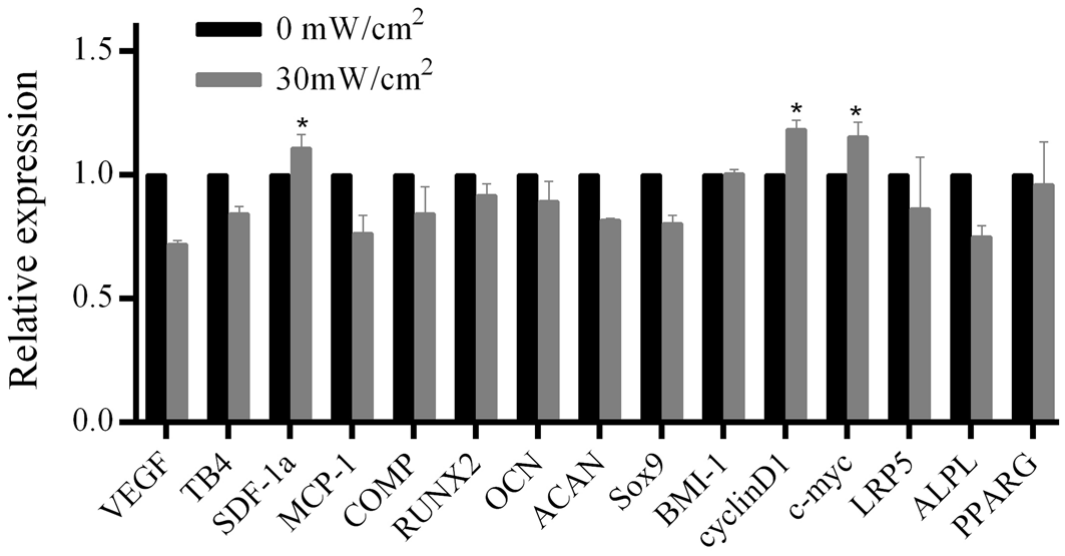
T The RT-PCR analysis results of the differentiated and functional genes with and without LIPUS stimulations. The experimental data of histograms were obtained by data processing the cycle threshold (CT) values of RT-PCR using the 2^-ΔΔCt^ method. The asterisks were the results of our data analysis using RT-PCR's CT values using two-independent sample t-tests.

The changes of LIPUS treated hASCs were analyzed in relative gene expression of osteogenesis, chondrogenesis, adipogenic differentiation, paracrine function, proliferation, and carcinogenic signaling pathway. hASCs cells in the stimulation group and control group were collected, and RT-PCR was used to detect osteogenic differentiation genes (*Runx2, OCN*), chondrogenic differentiation genes (*ACAN, COMP, SOX9*), adipogenic differentiation genes (*PPARG*), paracrine genes (*VEGF*, *TB4, McP-1*, and *SDF-1α*), proliferative signaling pathway genes (*CyclinD1, c-myc, LRP5*), and tumor-related genes (*BMI-1*), respectively. The results showed that *SDF-1α*, *CyclinD1,* and *c-myc* were up-regulated in the stimulation group compared with that of the control group (*p* < 0.05) (Fig. 5).

### Results of transcriptome experiments

To analyze the differences in transcriptional level of hASCs after LIPUS treatment, cells from the stimulation group and the control group were collected for transcriptome experiments.

#### Analysis of gene difference

**Figure 6:**
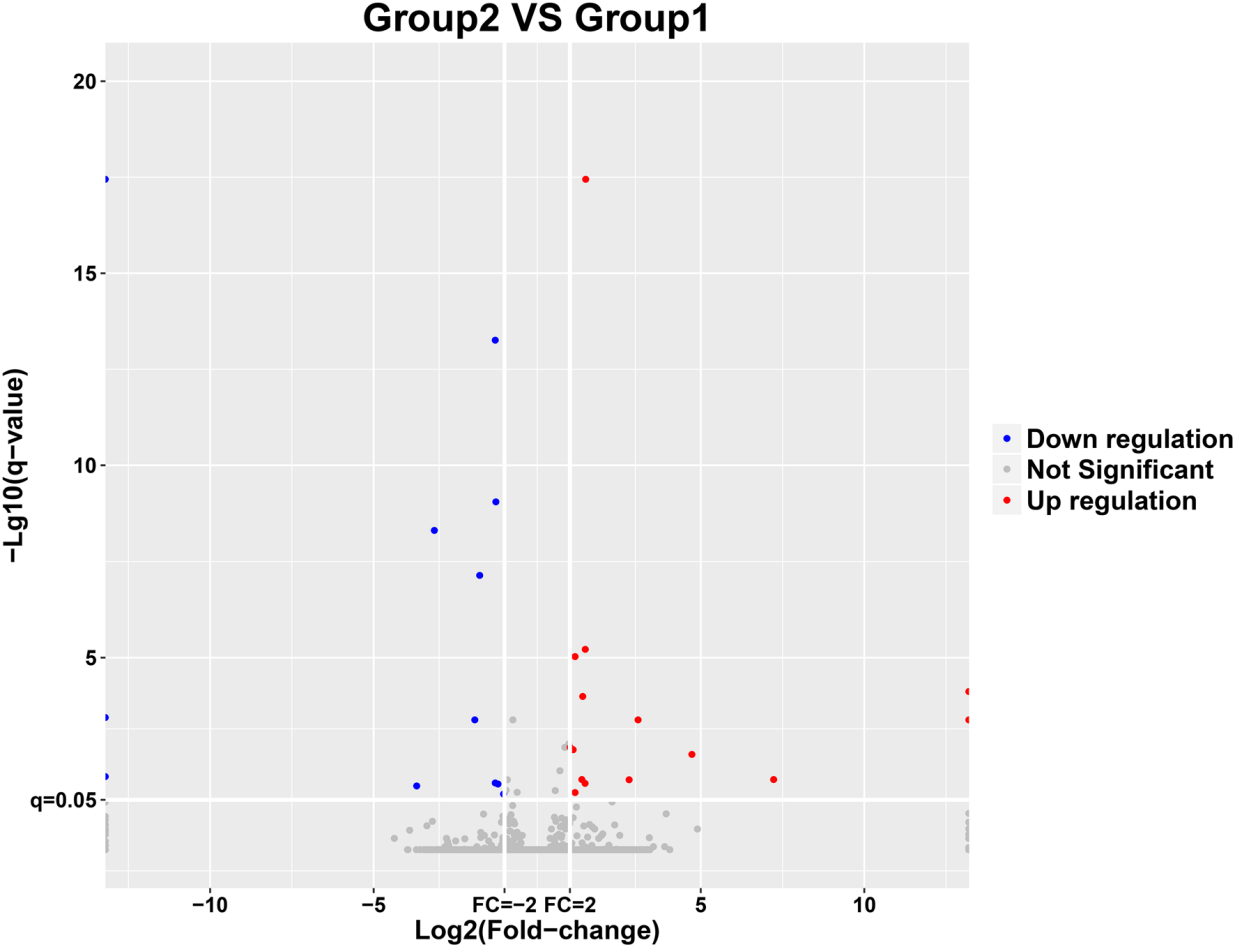

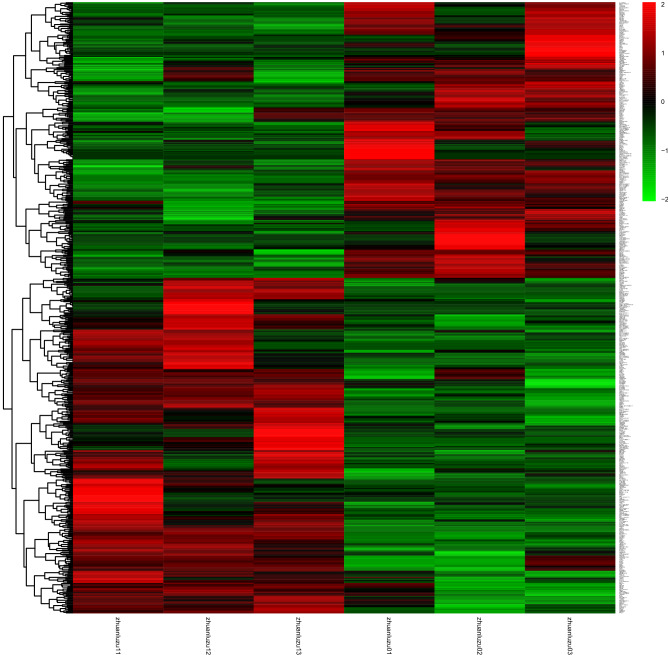
The transcriptome differential gene analysis of hASCs with and without LIPUS stimulation. EdgeR software was used to analyze the differential expression of genes. The differential expression multiples were clustered over the log2 conversion. The screening threshold of significantly differentially expressed genes was *p* < 0.05 and |log2 (FC) |≥ 1. (**a**) The volcano Plot of significantly differentially expressed genes (blue-colored dots are down-regulated genes while red-colored dots are up-regulated genes). Group 1 was the control group; group 2 was the LIPUS stimulation group. (**b**) The heat map of significantly and differentially expressed genes of hASCs with and without LIPUS stimulation. The stimulation group (zhuanlu11, zhuanlu12 and zhuanlu13) and the control group (zhuanlu01, zhuanlu02, and zhuanlu03).

EdgeR software was used to analyze the differential expression of genes. First, relative expression volume was generated based on the number of original reads of the gene in units of CPM (count per million). Then the differentially expressed genes were calculated according to their grouping. The differential expression multiples were clustered over the log2 conversion. The screening threshold of significantly differentially expressed genes was *p* < 0.05 and |log2 (FC) |≥ 1. Figure 6a shows the volcano map of significantly differentially expressed genes, and Fig. 6b shows the heat map of significantly differentially expressed genes. The results of differentially expressed genes showed that after treatment by LIPUS, 15 genes in the stimulus group were up-regulated (*KMT2A, CLCN5, AFF1, INO80B-WBP1, ZNF260, APC, CTD-3074O7.11, ZKSCAN8, ZNF267, AC006011.4, RP11-111K18.1, RP11-158I13.2, KB-1572G7.2, DYRK1A,* and *REL*), while 12 genes (*RP1-37E16.12, AHRR, ZNF512, PCDHGC4, SPIN1, BLOC1S5-TXNDC5, PPT2-EGFL8, TRIM39-RPP21, WFS1, NIPA1, DNAAF3,* and *YTHDF3)* were down-regulated.

#### GO pathway and KEGG pathway annotation and enrichment analysis of differential genes

**Figure 7:**
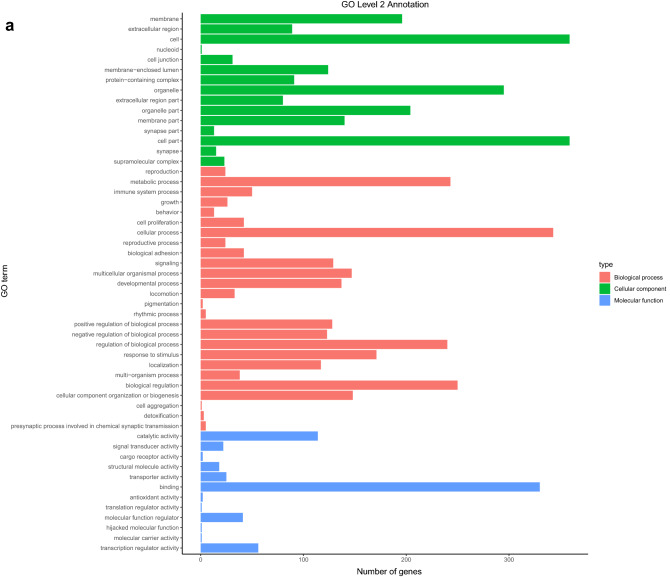

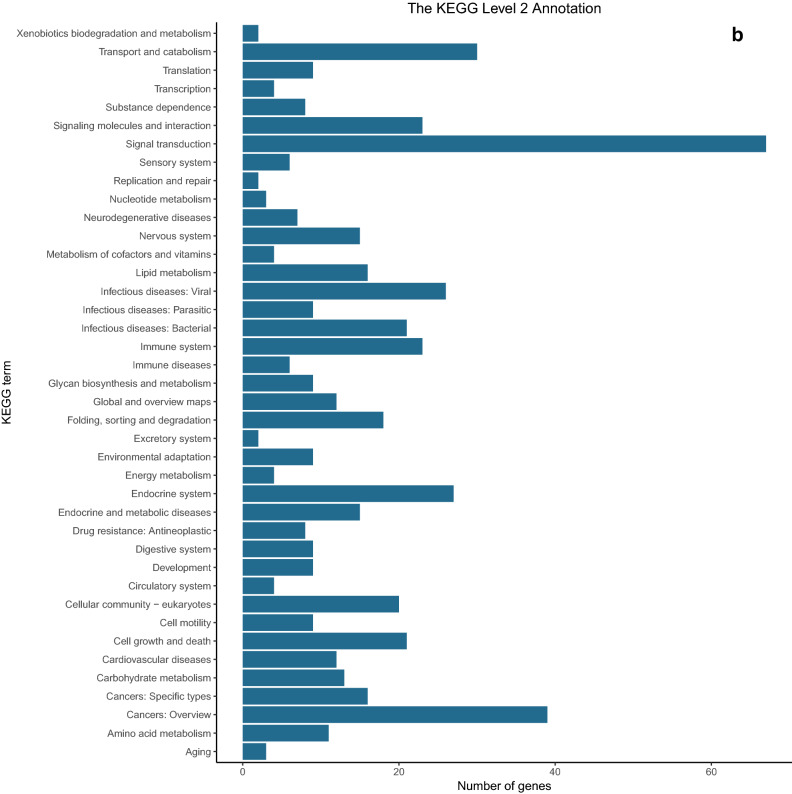
Gene difference analysis and enrichment between with and without LIPUS stimulations. The main pathways at the GO 2 and KEGG 2 level were annotated, and bar charts were drawn. (**a**) The GO analysis main concentration in cell components, biological processes, and molecular functions; (**b**) KEGG enrichment analyses of the different genes were mainly concentrated in signal transduction, transport, catabolism, and endocrine system of functions related to cell proliferation.

The significance (q-value < 0.05) results from the group difference gene comparison were used to annotate and enrich by the gene ontology (GO) and the Kyoto Encyclopedia of Genes and Genomes (KEGG). Annotation and enrichment analysis was performed using the R language clusterProfiler extension package and FDR method for multiple false-positive corrections. The significantly enriched GO and KEGG pathways were labeled in the form of bubble maps, and directed acyclic maps were drawn. At the same time, the main pathways at the GO 2 and KEGG 2 level were annotated, and bar charts were drawn. The results of GO analysis main concentrations were in cell components (including cell–cell, cellular components, organelles, and the cell membrane, etc.), biological processes (cells, metabolic process, many cell biological processes, development process, cell signaling, and cell proliferation, etc.) and molecular functions (molecular binding, catalytic activity and the activity of transcription regulation, etc.) (Fig. 7a). KEGG analysis showed that the related genes were mainly concentrated in signal transduction, transport, catabolism, and endocrine system of functions related to cell proliferation (Fig. 7b).

### Results of metabolome experiments

In this experiment, the data from 7 QC samples were analyzed and evaluated using two methods: total ion flow spectrogram comparison and principal component analysis. When the peaks were obtained from the experimental samples, QC samples were extracted, and PCA analysis was performed after auto-scaling. The QC samples, as shown in the PCA score graph, were clustered together, indicating excellent repeatability of the experiment in this study (n = 6).

#### Screening of difference indicators

Screening results of difference indexes: in this study, P value < 0.05, fold change > 2 times, and VIP value > 1 were selected as the screening indexes of different metabolic indexes (Table 3).

**Table 3:** Screening results of different indexes between stimulation group and control group.

Alignment ID	Average Rt(min)	Average Mz	Metabolite name	raw.pval	VIP	FC	Up/down
248	8.34	438.1955	methyl (1R,4aS,8S,8aS)-3-methoxy-1-methyl-8-[(2S,3R,4S,5S,6R)-3,4,5-trihydroxy-6-(hydroxymethyl)oxan-2-yl]oxy-1,3,4,4a,8,8a-hexahydropyrano[3,4-c]pyran-5-carboxylate	0.017334	1.6905	0.26983	Down
103	8.35	415.2119	2H-Oxireno[1,10a]phenanthro[3,2-b]furan-10(11bH)-one, 5,7-bis(acetyloxy)-3,3a,4,5,6,7,7a,7b,8,8a-decahydro-4,4,7a,11-tetramethyl-, (1aS,3aR,5S,7S,7aR,7bS,8aR,11bR)-	0.028625	1.9259	0.28659	Down
242	9.04	437.1952	5-hydroxy-7-[4-hydroxy-2-methoxy-3-(3-methylbut-2-enyl)phenyl]-2,2-dimethyl-7,8-dihydropyrano[3,2-g]chromen-6-one	0.0061098	1.7718	0.33901	Down
74	8.01	445.1865	Estrone-3-(beta-D-glucuronide)	0.022078	1.7526	0.34477	Down
28	8	404.2041	Cys His Lys	0.011739	1.7918	0.34694	Down
810	8.01	267.1204	S-Acetyldihydrolipoamide-E	0.0084697	1.3985	0.36358	Down
219	8.76	432.2385	7b,9-Dihydroxy-3-(hydroxymethyl)-1,1,6,8-tetramethyl-5-oxo-1,1a,1b,4,4a,5,7a,7b,8,9-decahydro-9aH-cyclopropa[3,4]benzo[1,2-e]azulen-9a-yl acetate	0.021196	1.5911	0.3681	Down
1,016	8.01	281.1021	Aspartylphenylalanine	0.0062387	1.7575	0.36815	Down
104	8.76	415.212	2H-Oxireno[1,10a]phenanthro[3,2-b]furan-10(11bH)-one, 5,7-bis(acetyloxy)-3,3a,4,5,6,7,7a,7b,8,8a-decahydro-4,4,7a,11-tetramethyl-, (1aS,3aR,5S,7S,7aR,7bS,8aR,11bR)-	0.025713	1.979	0.37448	Down
24	8.01	105.0663	L-2,3-DIAMINOPROPIONIC ACID	0.0087452	1.6959	0.37787	Down
87	8.01	413.174	2-Propenoic acid, 3-(4-hydroxyphenyl)-, 3-(beta-D-glucopyranosyloxy)-1-methylbutyl ester, (2E)-	0.018996	1.8949	0.39296	Down
148	8	121.0623	Phenylacetaldehyde	0.0054201	1.6709	0.40013	Down
1,216	8.76	295.1173	3-(2,4-dihydroxypentyl)-8-hydroxy-6-methoxyisochromen-1-one	0.025858	1.8575	0.40155	Down
1,019	8.76	281.1384	4-[5-(1,2-dihydroxypropan-2-yl)-2-methyloxolan-2-yl]benzoic acid	0.030417	1.6925	0.40403	Down
248	8.76	135.0802	Cinnamyl alcohol	0.025346	1.581	0.41574	Down
373	8.76	460.2699	17-phenyl trinor Prostaglandin E2 serinol amide	0.020178	1.7664	0.42984	Down
216	8	432.2372	Ala Trp Arg	0.011882	1.4408	0.43755	Down
241	8.76	437.1943	His Lys Met	0.019471	1.5631	0.44093	Down
2052	8.01	369.1695	(2S)-8-[(E)-3-hydroxy-3-methylbut-1-enyl]-5,7-dimethoxy-2-phenyl-2,3-dihydrochromen-4-one	0.034094	1.8768	0.44459	Down
71	8.01	410.1671	FucGlcNAcGA	0.0095403	1.7086	0.45437	Down
2079	6.31	371.2296	1-epi-Fortimicin B	0.038559	1.5223	0.4766	Down
878	9.76	604.3845	6,8a-Seco-6,8a-deoxy-5-oxoavermectin ''2a'' aglycone	0.00023391	1.418	0.47879	Down
366	8.76	459.2473	2-[(8,8-dimethyl-2-oxo-4-propyl-9,10-dihydropyrano[2,3-h]chromen-5-yl)oxy]-N-(2-morpholin-4-ylethyl)acetamide	0.02305	2.0566	0.49474	Down
998	7.52	279.0933	Triphenylphosphine oxid	0.028401	1.6577	2.2181	Up
401	4.76	305.0604	Gallocatechin	0.004652	1.7038	2.3711	Up
490	4.76	341.0385	Fungitox	0.0080018	1.7555	2.6258	Up
1,214	7.48	295.0868	Benzenemethanol, 2-chloro-alpha,alpha-diphenyl-	0.034315	1.652	2.9673	Up
975	7.36	277.0776	Propanamide, 2-methyl-N-[4-nitro-3-(trifluoromethyl)phenyl]-	0.034769	1.717	4.8279	Up
739	7.52	557.1802	(2E)-3-[4-(beta-D-Glucopyranosyloxy)-3,5-dimethoxyphenyl]-2-propen-1-yl beta-D-glucopyranoside	0.024932	1.4574	5.9085	Up
242	0.82	224.0866	1-(4-fluorophenyl)isoquinoline	0.041955	2.0688	31.446	Up

**Figure 8:**
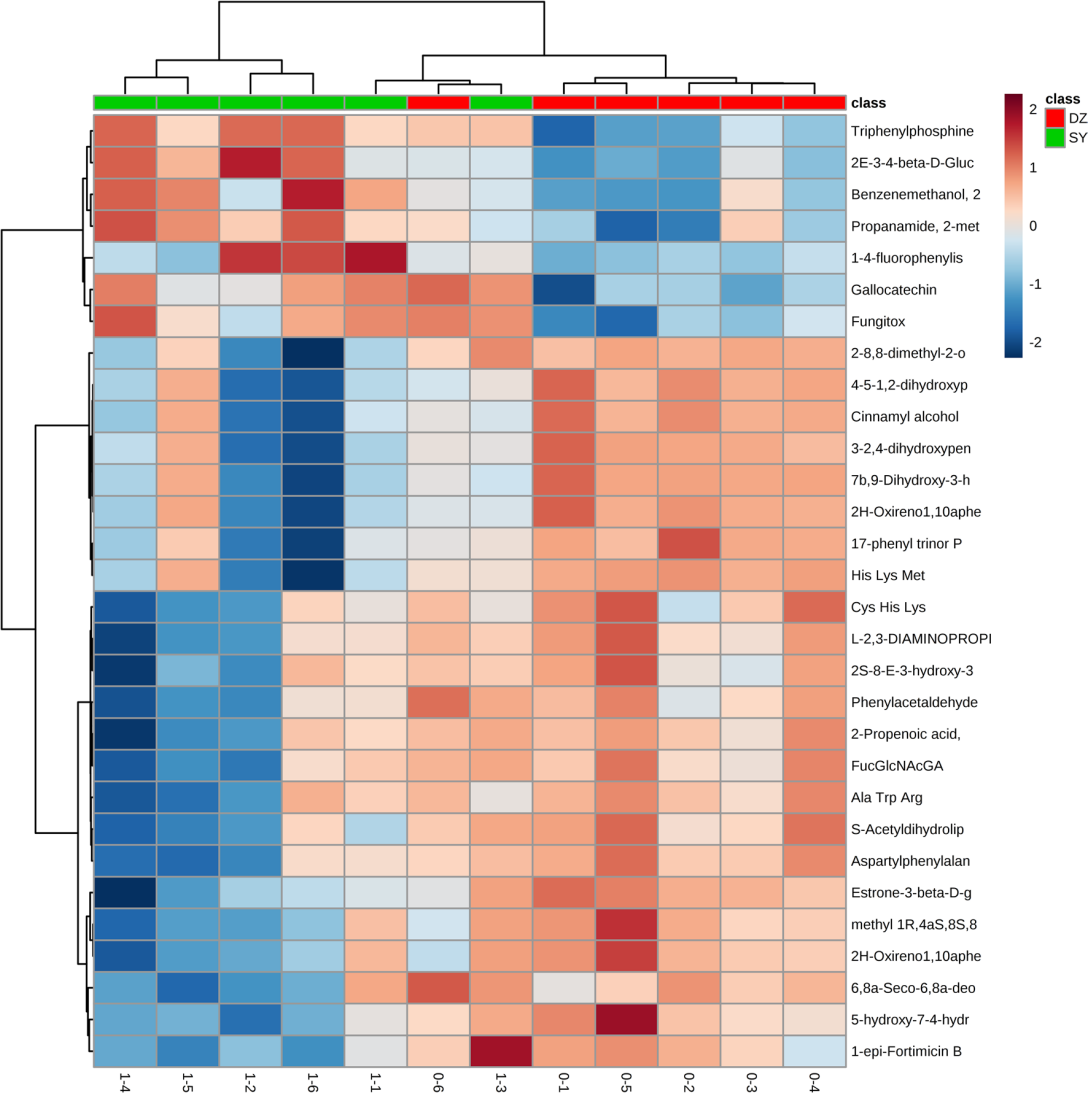
The results of different metabolites in terms of hierarchical clustering between stimulus group (SY) and control group (DZ). Hierarchical clustering was carried out for each group of samples using the expression amount of qualitatively and significantly different metabolites. The samples of the stimulus group and the control group can appear in the same cluster through clustering.

Cluster analysis of difference indicators: Hierarchical clustering was carried out for each group of samples using the expression amount of qualitative significantly different metabolites. As shown in Fig. 8, the samples of the stimulus group and the samples of the control group can appear in the same Cluster through clustering. Therefore, it is speculated that the metabolites clustered in the same cluster have similar expression patterns and maybe in relatively close reaction steps in the metabolic process.

#### Bioinformatics analysis

Metabolic pathway analysis: The different metabolites obtained by the stimulus group and the control groups were submitted to the online website https://metaboanalyst.ca to utilize their metaboanalyst 4.0 function for relevant pathway analysis, to understand which metabolic pathways significantly changed under the experimental conditions. Pathway analysis is a path analysis based on KEGG metabolic pathways, and univariate analysis, network topology analysis, and other novel algorithms are integrated.

1. Figure 9a,b show the metabolic pathways involved in the stimulus group and the control group.

**Figure 9:**
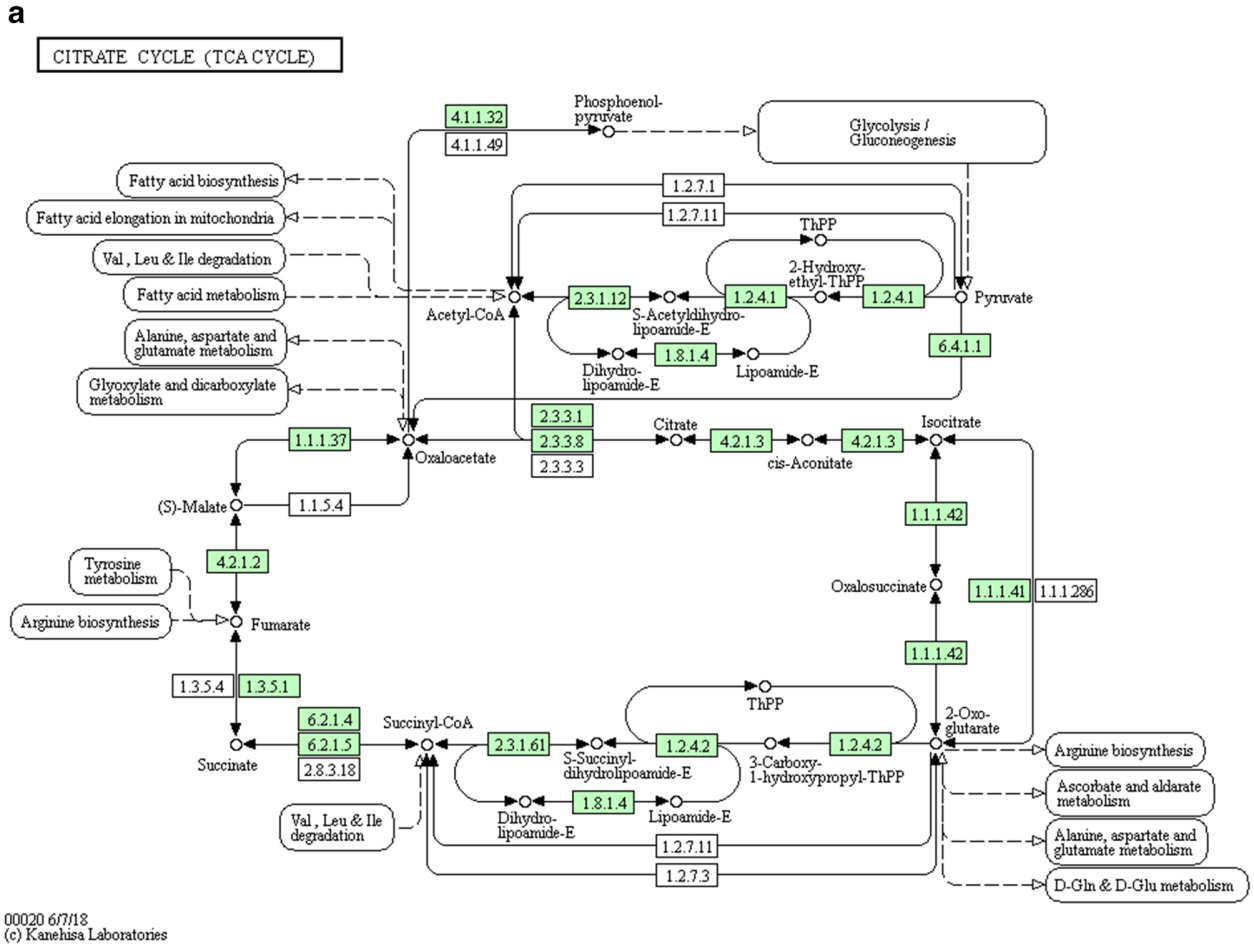

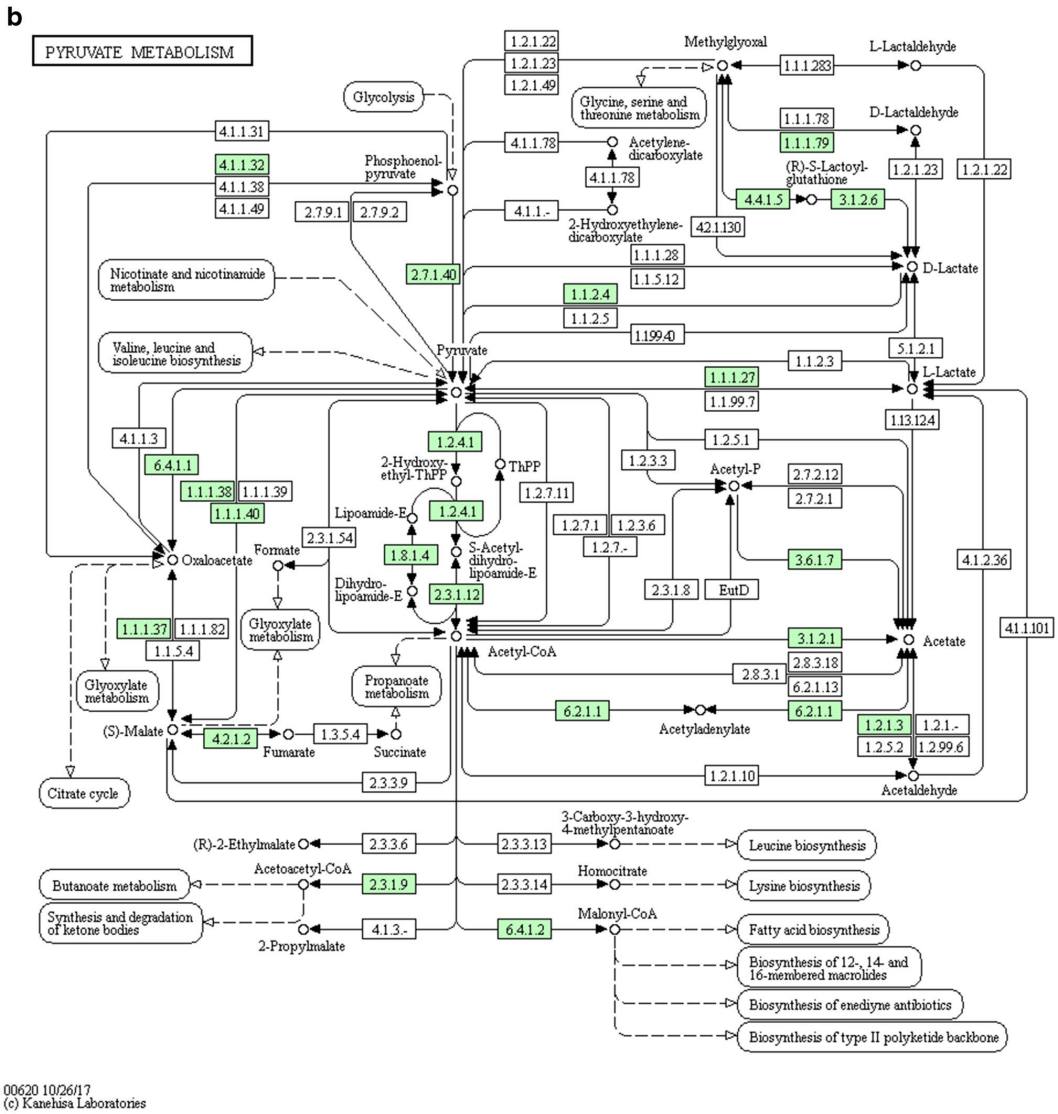
The pathway analysis of metabolic differences between the stimulus group and the control group. Different metabolites obtained by the stimulus group and the control groups were submitted to the online website metaboanalyst 4.0 for relevant pathway analysis. The metabolic pathways involved in the stimulus group and the control group was shown in Citrate cycle (TCA cycle) (**a**) and Pyruvate metabolism (**b**).
2. Figure. 10 shows the results of the pathway analysis in the form of a bubble diagram, where each bubble represents a metabolic pathway. The abscise-coordinate of the bubble and the size of the bubble represent the size of the pathway's influence factor in the topological analysis. The ordinate of the bubble’s location, and the color of the bubble represent the P-value of the enrichment analysis (negative natural logarithm, i.e., − log (P)). Darker color corresponds to a smaller P-value was, and the more significant degree of enrichment. The results showed that the Citrate cycle (TCA cycle) and Pyruvate metabolism had a higher influence value, suggesting that the metabolic pathway was significantly changed.

**Figure 10:**
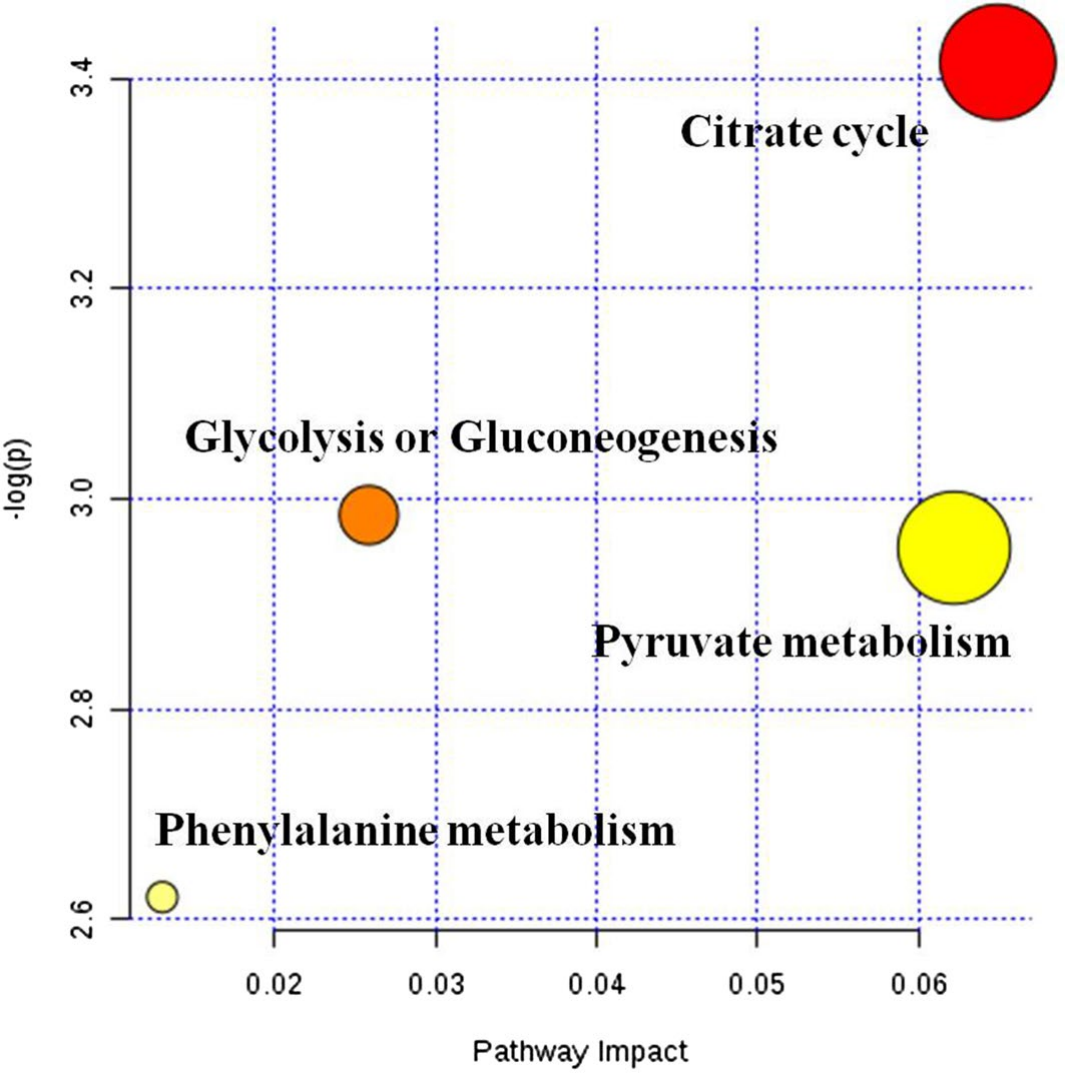
The summary and analysis of metabolic difference between the stimulation group and the control group. In the bubble diagram, each bubble represents a metabolic pathway. The ordinate, where the bubble was, and the color of the bubble represent the *p *value of the enrichment analysis (i.e., −log (P)). The darker the color was, the smaller the *p *value was, and the more significant the enrichment degree was. The Citrate cycle (TCA cycle) and Pyruvate metabolism had a higher influence value, which might be the significantly changed metabolic pathway.

**Figure 11:**
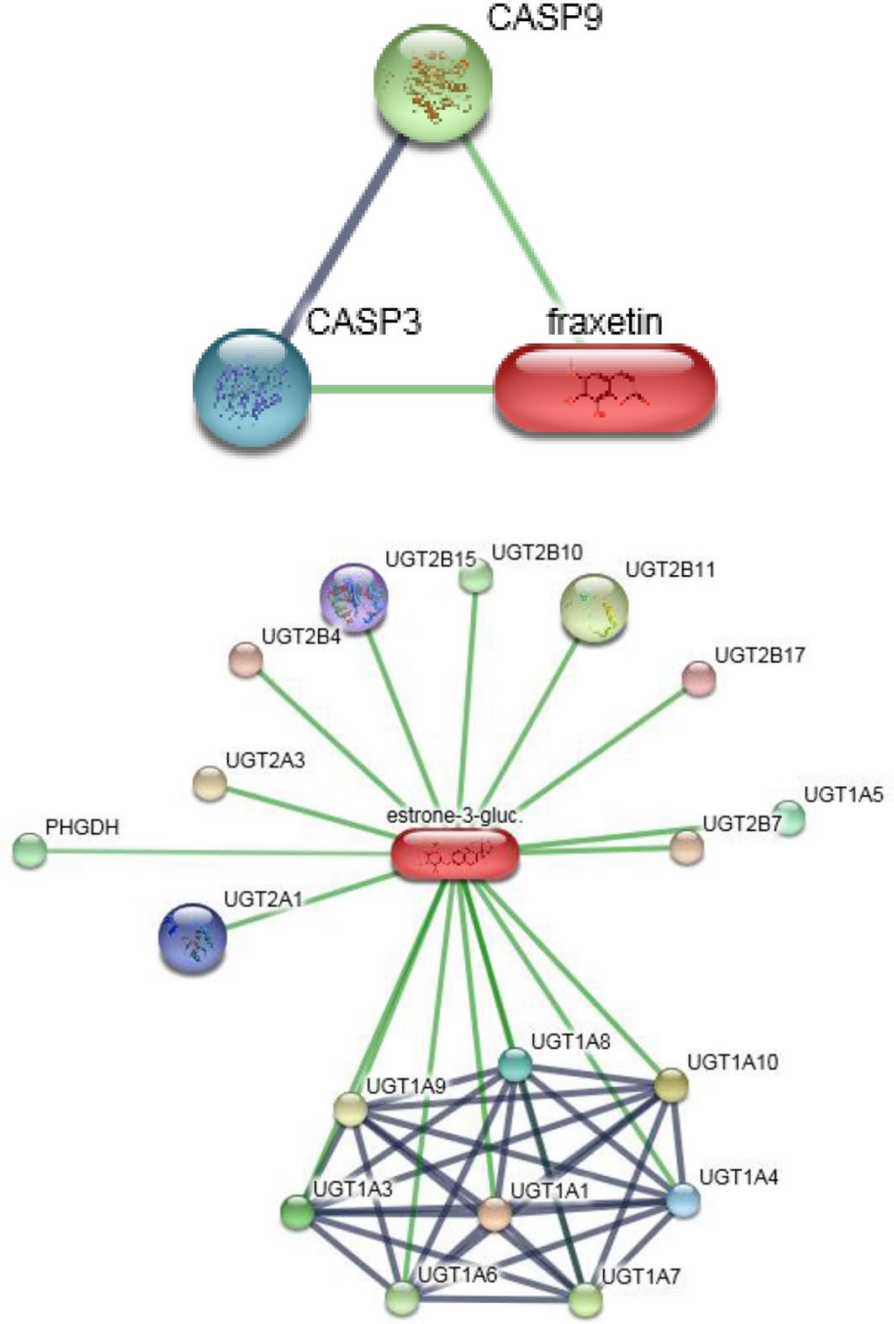
Genes of interactive metabolites. To search for genes that interacted with metabolites, the selected metabolite names were searched in the stitch database for the genes interacting with them. Fraxetin (left) and estrone 3-glucuronide (right) were found the genes that interacted in the stitch database.

To search for genes that interacted with metabolites, the selected metabolite names were searched in the stitch database (https://stitch.embl.de/) for the genes interacting with them. Among these metabolites, the two differentially expressed metabolites (fraxetin and estrone 3-glucuronide) were found to have interacting genes in the stitch database (Fig. 11).

## Discussions

Our study showed that LIPUS could promote the proliferation of hASCs. We compared the effects of LIPUS of differening intensities on hASCs proliferation. The results showed that 30 mW/cm^2^ and 5 min/day was the best stimulation setting. Cell proliferation was significantly different after the second-time stimulation, and the promoted proliferation in different passages (P3, P6, and P8) was also observed. However, the proliferation of hASCs cells was inhibited by 10 min/day stimulation. The morphology of the cells after proliferation was consistent with that of mesenchymal stromal cells, which grew in a spindle and vortex shape^31,32^. After stimulation, the cell surface markers CD105, CD73, and HLA-ABC were positively expressed, while CD14, CD34, and CD45 were negatively expressed, which showed no significant difference compared with the cell surface markers of the control group. The cell surface markers were accorded with the characteristics of mesenchymal stromal cells and were consistent with previous studies^33,34^.

hASCs can regulate the fate of T cells and their own immune function. It also shows obvious adaptability to environmental stress and secretes a variety of paracrine factors that promote tissue regeneration, which is expected to achieve successful immunotherapeutic effects and tissue repairability^10,35^. In our study, after LIPUS stimulations, the release capability of hASCs cytokines (IL-6, IL-2, FGF2, and EGF) in culture medium was enhanced, and the expression of paracrine gene *SDF-1α* was up-regulated. Additionally, the apoptosis rate was decreased, and the proliferation ability of cells was increased. These results suggest the enhanced functionality of hASCs after LIPUS processing.

Previous studies on LIPUS promoting different stem cell proliferation pathways have generally been consistent. Ling et al*.* used LIPUS to treat human amnion- derived mesenchymal stromal cells (hAD-MSCs). The results showed that cell proliferation plays a role through the ERK1/2 and PI3K-AKT signaling pathways^29^. Budhiraja et al*.* studied human bone-marrow-derived mesenchymal stromal cells (hMSCs) and showed that LIPUS promoted proliferation and self-renewal of hMSCs through the activation of MAPK/ERK and PI3K/AKT pathways^27^. Xie et al*.* studied human bone marrow stem cells (hBMSCs) and suggested that LIPUS exposure may be involved in the proliferation of hBMSCs through the activation of PI3K/AKT signal pathway^28^. Ren et al*.* studied Schwann cells and showed that LIPUS promoted the activity and proliferation of Schwann cells by enhancing GSK-3β/β-catenin signaling pathways^36^. Budhiraja et al*.*, Xie et al*.* and Ren et al*.* all believed that LIPUS promoted cell proliferation by up-regulating the expression of *CyclinD1*^27,28,36^*CyclinD1* is an early component of cell cycle signaling and binds to Cyclin-dependent Kinase 4 (CDK4), or Cyclin-dependent Kinase 6 (CDK6) to form active complexes. This complex leads to a sequence of transcriptional activation and expression of cell cycle signaling molecules that drive cells into the next phase of the cell cycle^36^. In our experiment, G1 and S phase of hASCs cell cycle were significantly increased, and the expression of *CyclinD1* gene was up-regulated after LIPUS stimulation, which was consistent with previous reports. Interestingly, *c-myc* gene expression was also up-regulated after LIPUS stimulation in our experiment. This was consistent with what Paula et al*.* reported^37^, human adipose tissue-derived stem cells cultured in the absence of exogenous culture conditions enhance *c-myc* gene expression and promote proliferation. Therefore, we hypothesized that the effect of LIPUS stimulation on the up-regulation of *CyclinD1* and *c-myc* gene expression and hASCs proliferation was due to the activation of various upstream signaling elements and the activation of MAPK/ERK and PI3K/AKT signaling pathways. First, the activated MAPK/ERK signaling pathway promotes the transcription of *CyclinD1,* a major regulator of cell proliferation, through phosphorylation, and helped hASCs complete the process of the cell cycle. Second, phosphorylated AKT promotes the expression of *c-myc* and myc-related factor X (MAX) form complexes that promote the transcription of *Cyclin-D1.* Finally, the hASC cell cycle was promoted from the G1 phase to the S phase, and cell proliferation was promoted as well. These results were similar to the cell proliferation model hypothesized by Budhiraja et al*.*^27^.

In previous studies, high-intensity LIPUS and prolonged stimulation promoted stem cell differentiation toward osteogenesis and adipogenesis formation^38–41^. However, in our study, LIPUS of 30 mW/cm^2^, stimulated for 5minutes/day, showed no significant difference in osteogenic, adipogenic, and chondrogenic differentiation genes and cell surface markers compared with that of the control group. This suggests that LIPUS of 30 mW/cm^2^ could promote the proliferation of hASCs cells without differentiation, while maintaining the characteristics of stem cells.

The transcriptome is the sum of all gene transcriptome probes in a specific tissue or cell, which is related to the genetic information of the genome and the biological function of the proteome. It is an effective means to study the molecular mechanism of different cell processes^25^. On the other hand, metabolomics is a new discipline that conducts a simultaneous qualitative and quantitative analysis of all low molecular weight metabolites of a particular organism or cell in a specific physiological period. It is a branch of system biology which is based on group index analysis, high throughput detection and data processing. Metabolomics aims at information modeling and system integration^42,43^. Metabolomics and transcriptome analysis improve our understanding of stem cell function and are more conducive to the analysis of metabolic pathways and transcriptional gene differences involved in the regulation of stem cell fate, including self-renewal, proliferation, and differentiation^25,44^. In our experiment, transcriptome analysis after LIPUS stimulation revealed significant differences in 27 genes, with 15 genes up-regulated and 12 down-regulated. Among them, *KMT2A, APC, DYRK1A,* and other genes that played an important role in transcriptional activation^45,46^, cell adhesion^47^, and regulation of cell proliferation signals, were up-regulated^48^. Meanwhile, WFS1 and other genes that participated in the occurrence and development of a variety of diseases, were down-regulated^49^.

The GO analysis revealed the primary pathways leading to enrichment of genetic variations in cell components (including cell–cell and cellular components, organelles, cell membrane, etc.), biological processes (cellular processes, metabolic processes, many cell biological processes, cell signaling, cell proliferation, etc.) and molecular functions (molecular binding, catalytic activity and the activity of transcription regulation, etc.). KEGG analysis showed that the differentially expressed genes were mainly concentrated in signal transduction, transport and catabolism, signal molecules and interaction, cell growth and death, and endocrine system of functions related to cell proliferation.

It has been reported that the Ras pathway, once activated, initiates the cascade amplification of serine—threonine kinases. It then recruits Raf-1 serine—threonine kinase from the cytoplasm to the cell membrane, where Raf kinase phosphorylates MAPK kinase (MAPKK, also known as MEK) and MAPKK activates MAPK (also known as ERK). MAPK is activated and then translocated into the cell nucleus to directly activate transcription factors. In addition, MAPK stimulates Fos and Jun transcription factor that forms the transcription factor AP-1, which binds to a specific DNA sequence next to the *myc* gene to initiate transcription. *myc* gene products are also transcription factors that activate other genes. Ultimately, these signals converge to induce Cyclin D expression and activity^50^. On the other hand, transcription factor nuclear factor κB (NF-κB) family members (RelA/p65, RelB, c-Rel, and p50/p105) up-regulate the Ras signaling pathway and its downstream gene expression^51^. Interestingly, in the present experiment, we enriched the Rel protein family-associated gene REL(c-Rel), suggesting that hASCs stimulated by LIPUS promote cell proliferation through activation of the Ras signaling pathway and downstream pathway gene expression. The finding is consistent with our hypothesis.

In the metabolomics experiment, 30 metabolites showed significant differences; 7 metabolites were up-regulated, and 23 metabolites were down-regulated. Bioinformatics analysis of metabolites showed that the Citrate cycle (TCA cycle) and Pyruvate metabolism had a higher influence value, which might indicate the metabolic pathway has significantly changed. Studies have reported that Citrate cycle and Pyruvate metabolism were closely related to mitochondrial metabolism, cell cycle and stem cell proliferation^52–54^. These two differential metabolites, Fraxetin and Estrone 3-glucuronide, were found to interact with genes in the stitch database, and both showed down-regulation effects. Fraxetin has been reported to inhibit cell proliferation, induce and inhibit anti-inflammatory and tumor development^55,56^. It was suggested that decreasing the expression of Fraxetin may promote cell proliferation. Estrone 3-glucuronide is closely related to UDP-uridine diphosphate glucuronosyltransferases (UGT) and is involved in UGT's function and material transport^57^. UGT also plays an important role in drug metabolism and detoxification^58^, which may indicate that LIPUS stimulation was less toxic to hASCs cells. Therefore, transcriptome and metabolome studies have shown that LIPUS stimulation of hASC cells promotes cell proliferation through differences in genes related to cell proliferation and significant changes in metabolites in metabolic pathways. The specific mechanism remains to be further studied.

Stem cells and their local microenvironment (or niche) use signals to regulate the fate and behavior of their cells when subjected to external mechanical forces. In vitro, synthetic models of stem cell niches can be used to precisely control and manipulate the biophysical and biochemical properties of the stem cell microenvironment and to guide stem cell differentiation and regeneration^15,16^. LIPUS has been widely applied as a non-invasive treatment method for the transmission of mechanical stimulation through the skin to biological tissues in the form of high-frequency, small amplitude, and pulse pressure waves^17,18^. At the cellular level, ultrasound has been reported to promote microbial growth by releasing cell bundles, increasing cell membrane permeability, regulating the medium, and effect on cell composition, cell function, and genetics. At the molecular level, ultrasound promotes or destroys enzyme activity by changing the properties of enzymes, substrates, the reaction between enzymes and substrates, and provides an optimal environment for the reaction^59^. Ultrasound treatment at an appropriate frequency and intensity level can induce favorable changes in the conformation of protein molecules without changing their structural integrity, thus improving the enzyme activity^60^. These results suggested that the application of LIPUS with the appropriate intensity will be beneficial to the development of stem cell proliferation and regenerative medicine.

Interestingly, both the PPT2-EGFL8 gene and the S-Acetyldihydrolipoamide-E metabolite were shown to be down-regulated in this experiment. PPT2-EGFL8 gene plays an active role in lipid acyl hydrolase activity and fatty acid metabolism^61^. Similarly, S-Acetyldihydrolipoamide-E is a chemical that supports the formation and maintenance of structured and functioning cell or organelle membranes, which, together with proteins and other lipids, maintain the stability of the membrane structure, thereby further strengthening the membrane and reducing its permeability. It acts directly or indirectly with acetyl coenzyme A. It is involved in cellular processes that synthesize or break down lipid molecules to provide energy or storage and plays a vital role in the TCA cycle and Pyruvate metabolism^62,63^ (Fig. 9). Therefore, we speculate that the PPT2-EGFL8 gene and S-Acetyldihydrolipoamide-E co-regulate the fatty acid metabolic pathway involved in LIPUS stimulation to promote cell proliferation. The exact mechanism is subject to further investigation.

In conclusion, LIPUS could promote the proliferation of hASCs. LIPUS may activate MAPK/ERK, and PI3K/AKT signaling pathways by up-regulating the *CyclinD1* gene and *c-myc* gene in hASCs cells without differentiation. The proliferation of cells was facilitated by the significantly different changes in multiple genes of the transcript and various products of metabolism. The results of this study provided important clues for the clinical application of LIPUS in promoting the proliferation of hASCs cells.

## Supplementary information

Supplementary file1

## Data Availability

The data that support the findings of this study are available from the corresponding author upon request.
