# Supplementary material for: Impact of low-intensity pulsed ultrasound on transcription and metabolite compositions in proliferation and functionalization of human adipose-derived mesenchymal stromal cells
Source: Sci Rep. 2020 Aug 13;10:13690. doi: 10.1038/s41598-020-69430-z (PMC7426954; doi:10.1038/s41598-020-69430-z)
Supplement: Supplementary file 1 — Supplementary file1 [file 41598_2020_69430_MOESM1_ESM.docx]

**Supplementary Information:**

**Impact of Low-intensity Pulsed Ultrasound on Transcription and Metabolite Compositions in Proliferation and Functionalization of Human Adipose-derived Mesenchymal Stromal Cells**

Denggao Huang, Yuanhui Gao, Shunlan Wang, Wei Zhang, Hui Cao, Linlin Zheng, Yang Chen, Shufang Zhang, Jie Chen

**Supplementary Figure 1:** The analysis of hASCs in the stimulation group and the control group using the Ki-67 cell proliferation assay. Ki-67 antibody was used in immunofluorescence (IF) experiments. The cell nucleuses were stained by 4', 6-Diamidino-2-Phenylindole, Dihydrochloride (DAPI). Yellow arrows point to red spots, indicating positive Ki-67 expression. Scale bar was 100 µm.

**
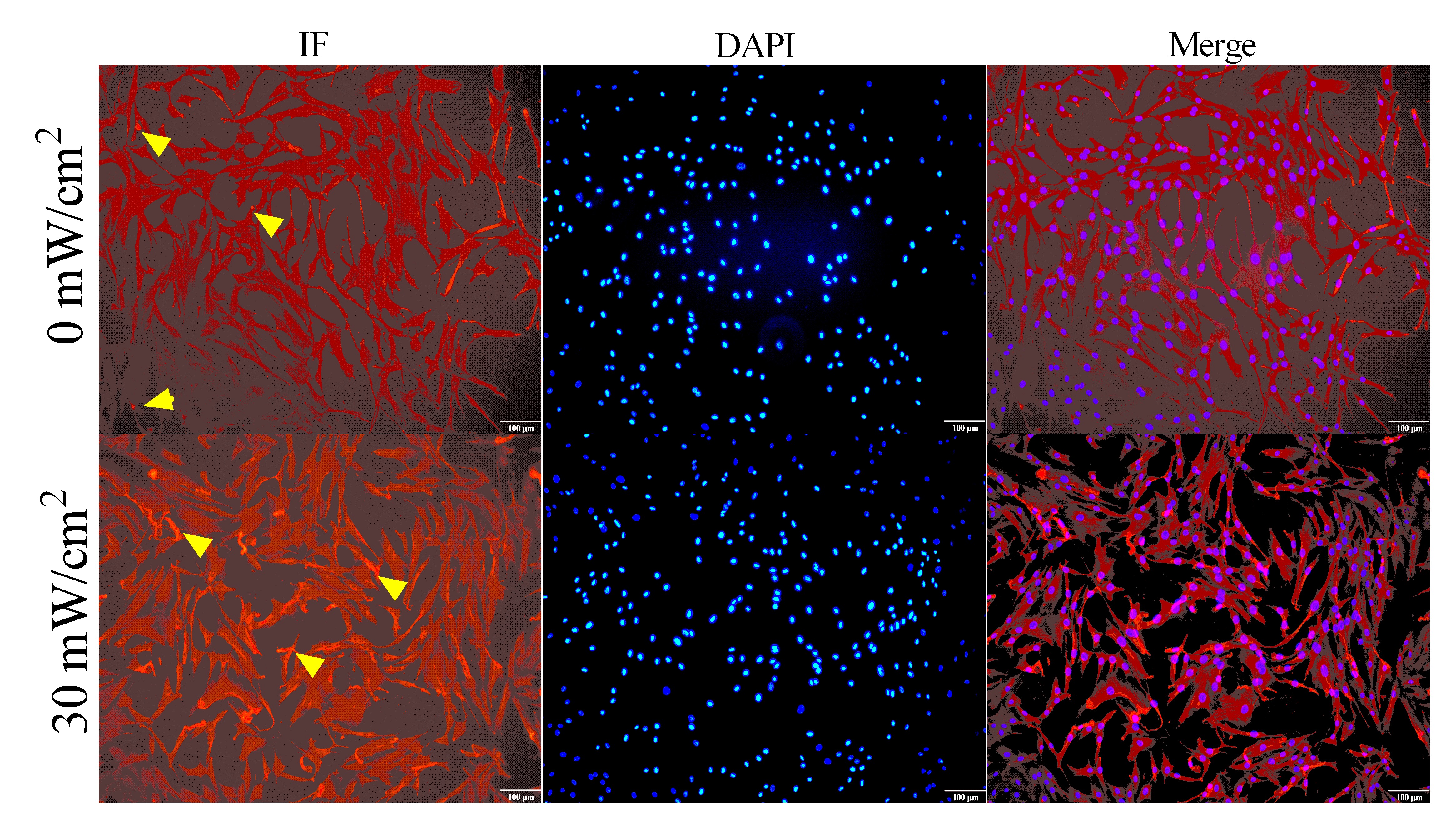
**

**Supplementary Figure 2:** Gate plot and scatter plot of Flow cytometry (FCM) results. The data analysis is related to the supplementary table.


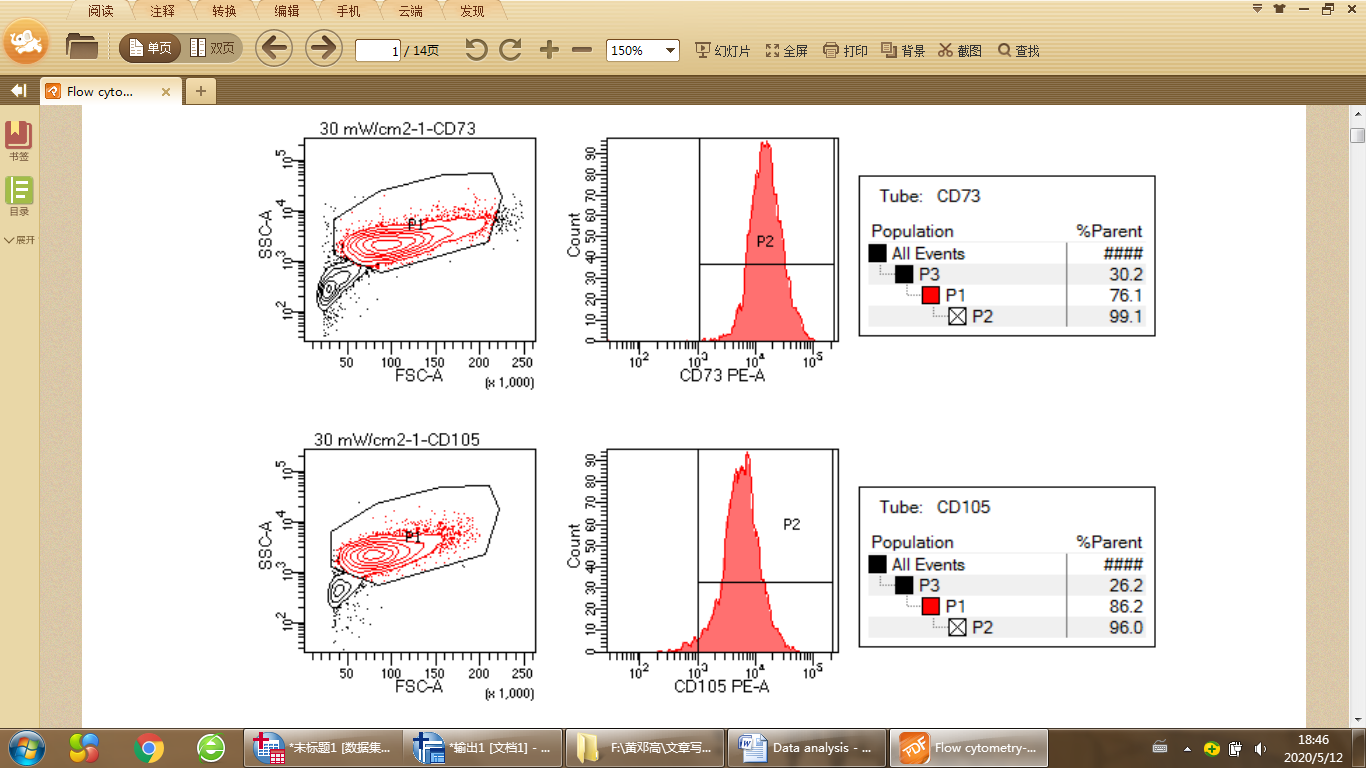


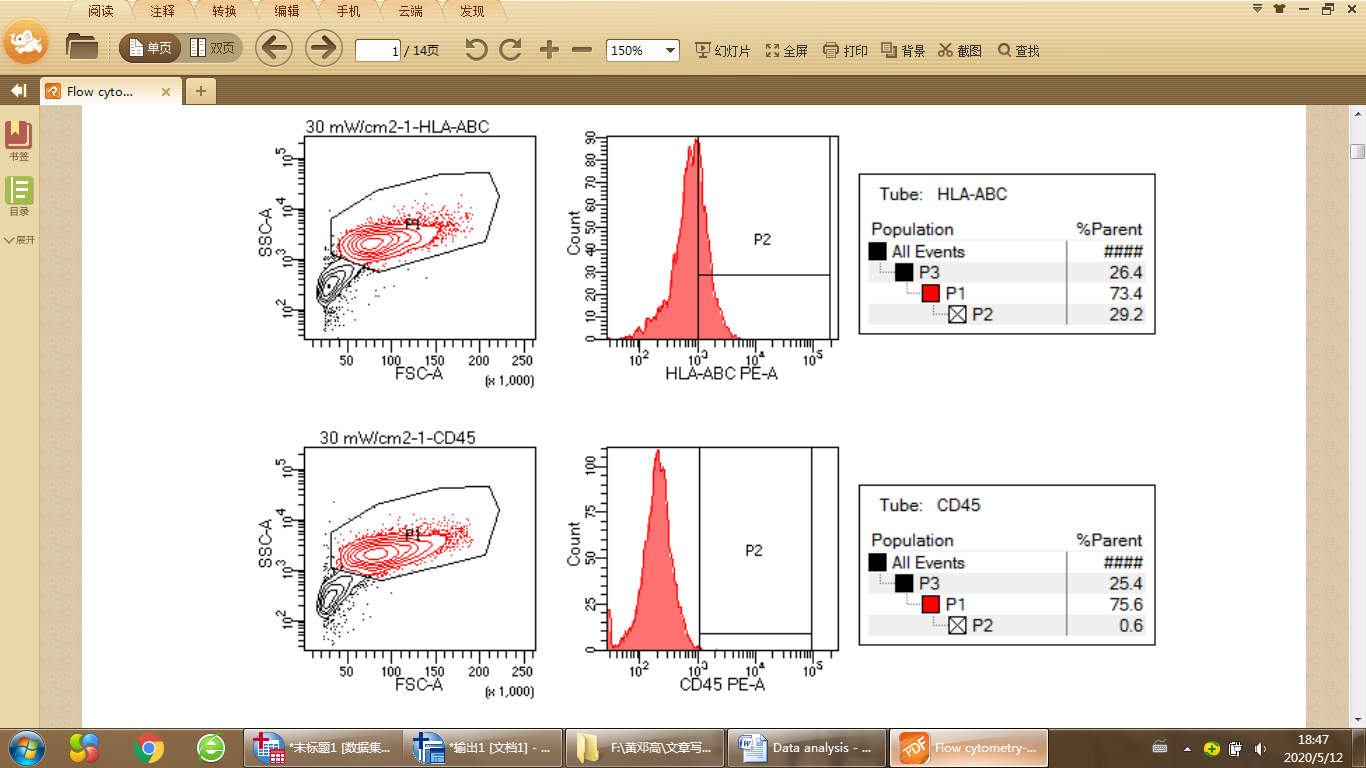


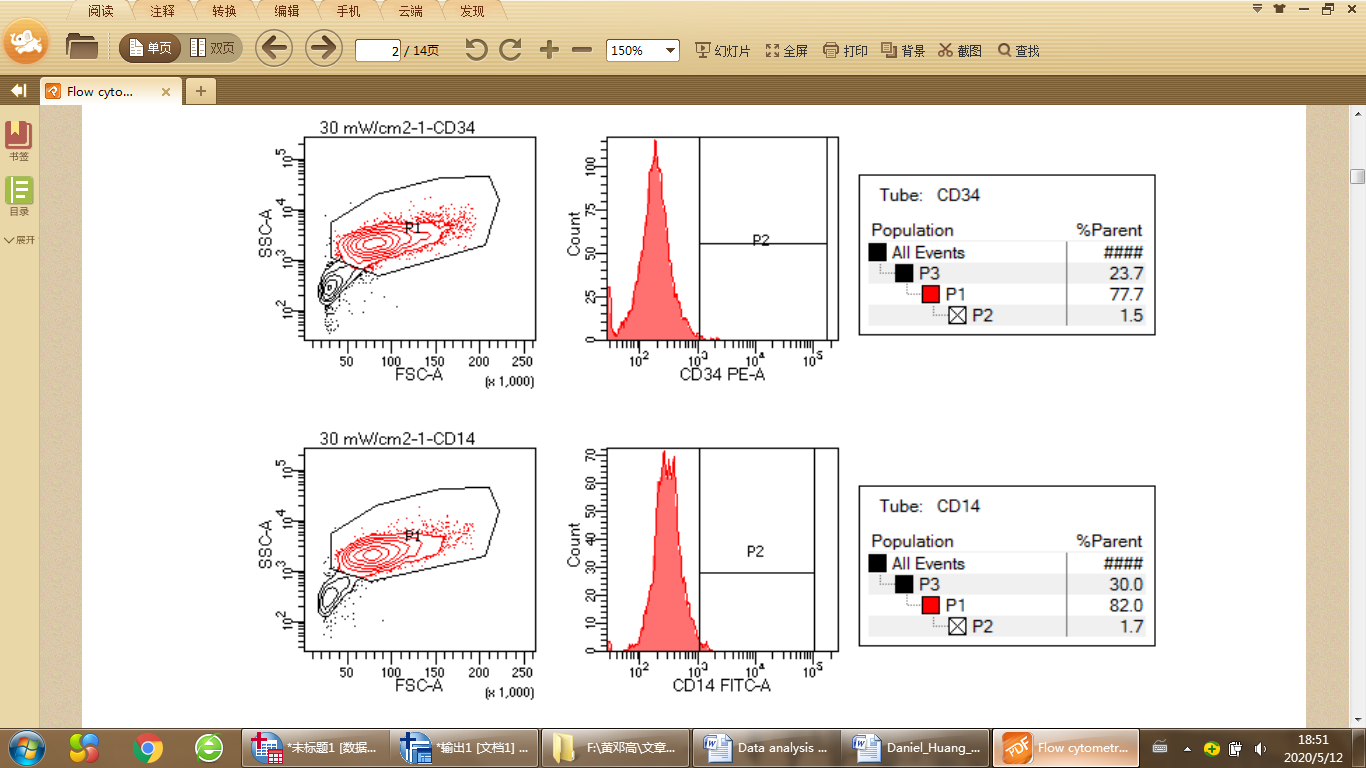


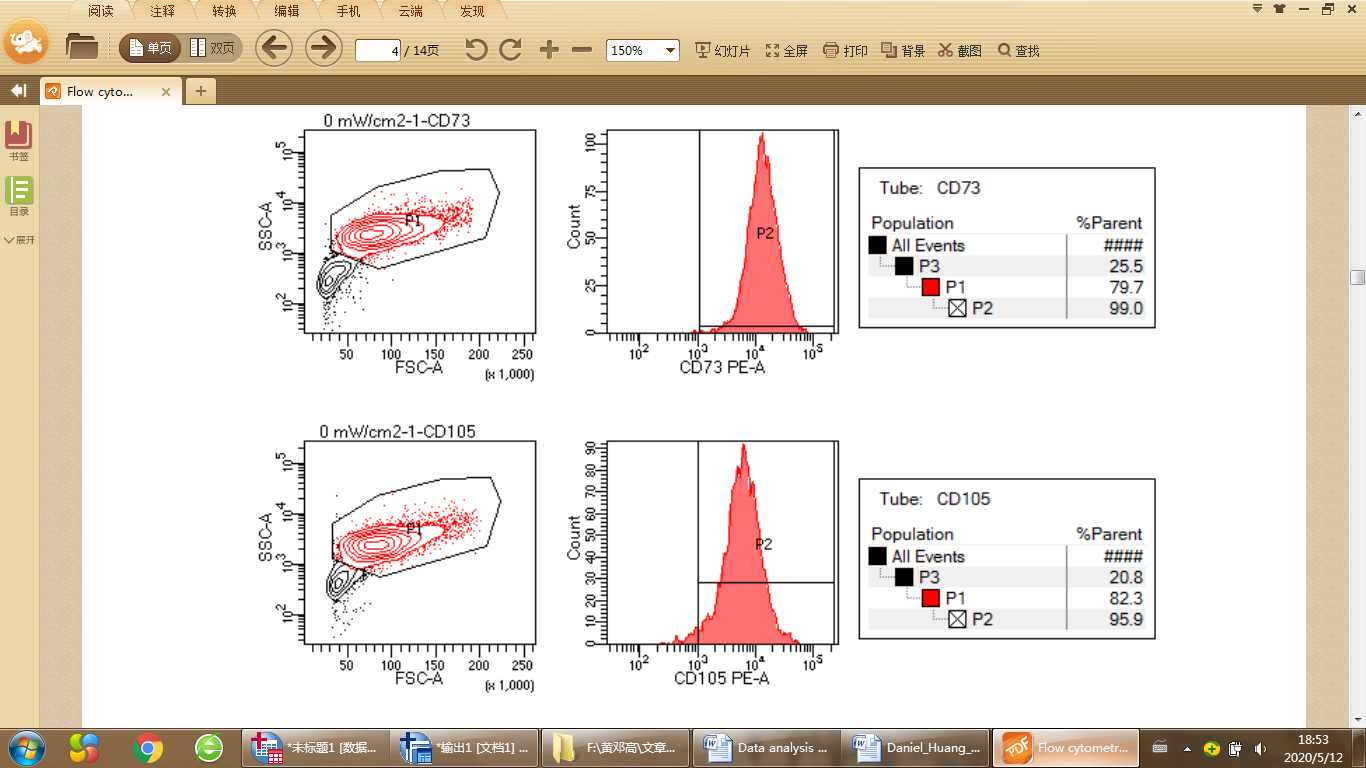


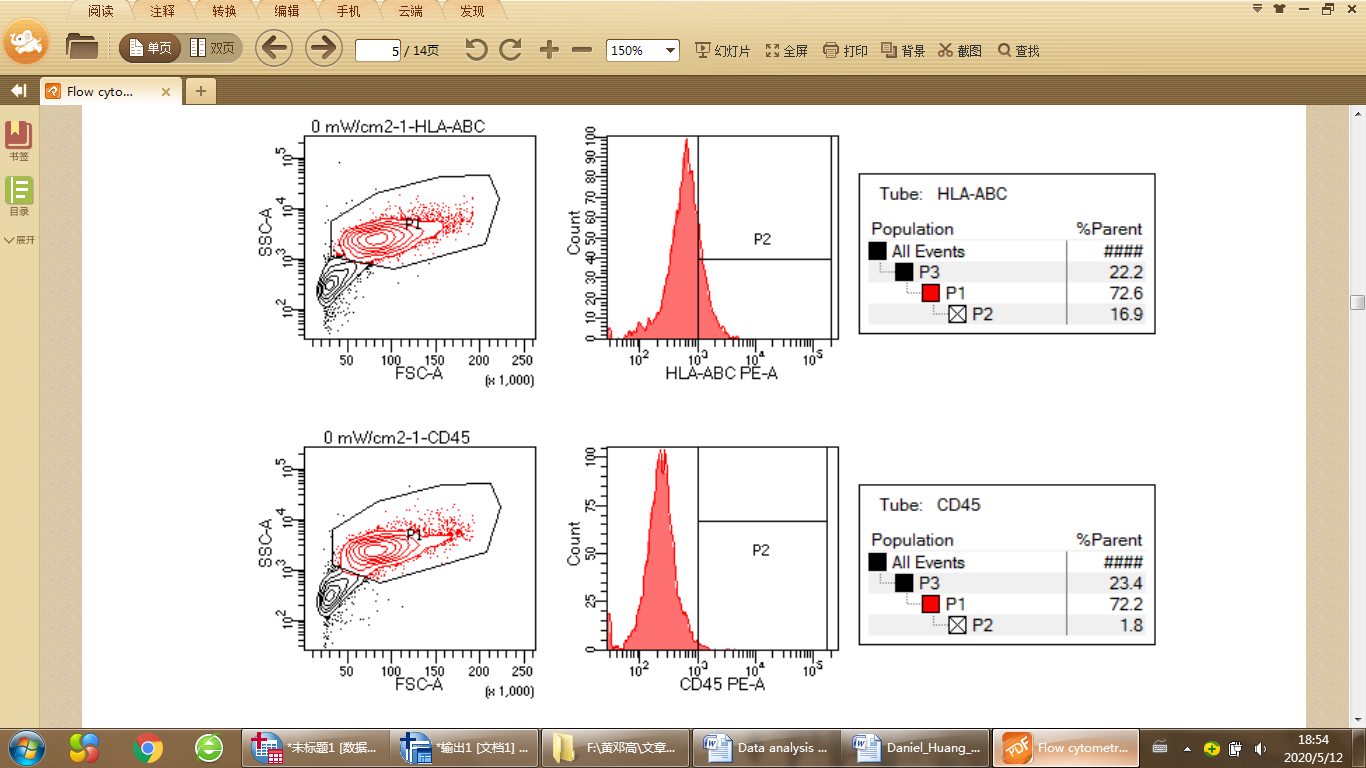


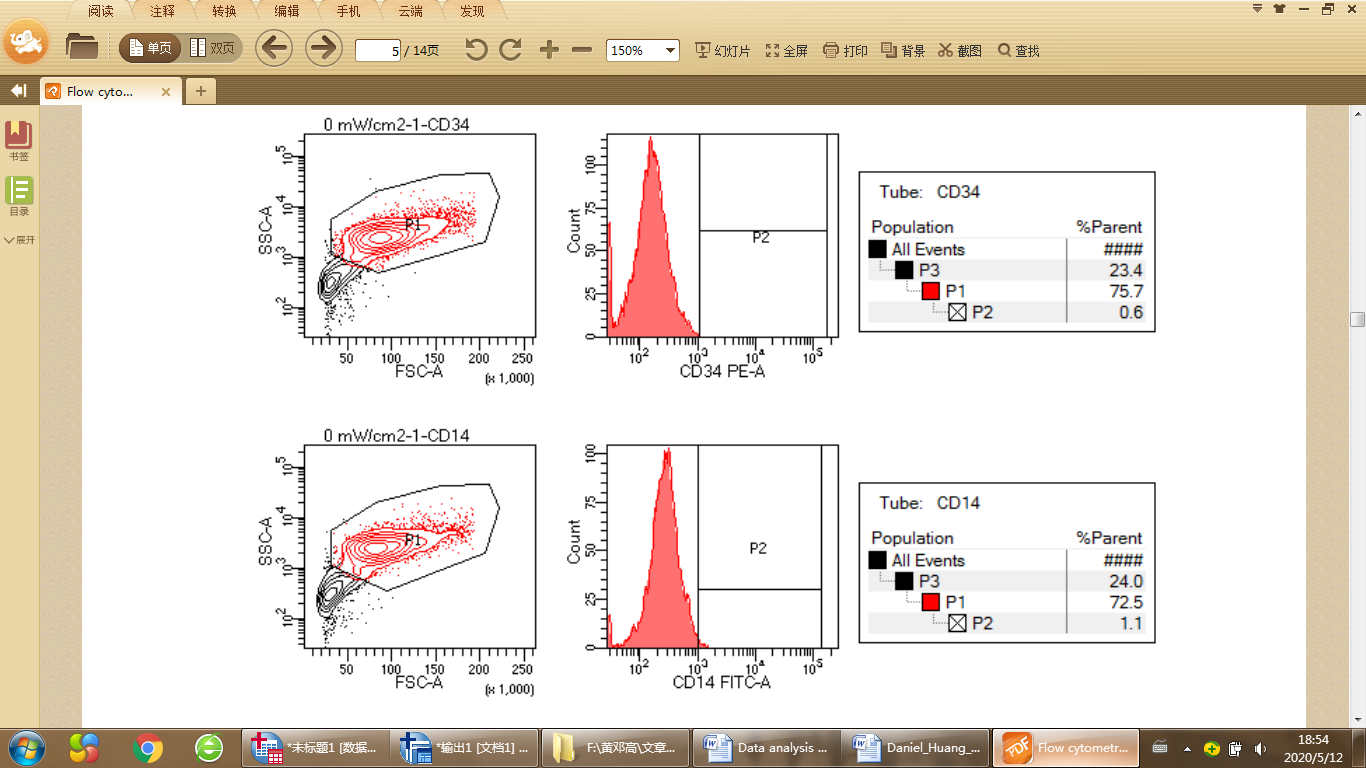


**Supplementary Table:** Comparison of hASCs cell surface markers between the stimulation group and the control group (P8, n=3).

|  | CD73 | CD105 | HLA-ABC | CD45 | CD34 | CD14 |
| --- | --- | --- | --- | --- | --- | --- |
| 0mW/cm^2^ | 98.97±0.25 | 95.33±0.81 | 22.07±5.59 | 1.33±0.57 | 0.97±0.35 | 0.97±0.71 |
| 30mW/cm^2^ | 99.07±0.15 | 95.13±0.96 | 23.50±5.23 | 0.93±0.35 | 1.13±0.40 | 1.23±0.45 |

The expressions of hASCs surface markers CD105-PE, CD73-PE, HLA-ABC-PE, CD14-FITC, CD34-PE, and CD45-PE were determined by flow cytometry in the stimulation group and the control group. No difference between stimulus and control group data. The experimental data was statistically analyzed using two independent sample t-tests. Means ± standard deviation (SD) were used to present the quantitative data.

**Supplementary experiment protocol:** the Ki-67 cell proliferation assay

The cells were fixed with freshly prepared fixation solution (4% Paraformaldehyde in PBS, pH 7.4) at room temperature (RT) for 45-60 min, and then rinsed them 3 times with PBS. After that, the cells were incubated in permeabilization solution (0.1% Triton X-100 in 0.1% fresh sodium citrate) at RT for 5-10 min and rinsed cells 3 times with PBS, then incubated in blocking buffer at RT for 30-45 min. Subsequently, the anti-Ki67 rabbit antibody (1:250) was added and incubated at 4 ℃ in a humidified atmosphere overnight. The next day brought the cells from 4 ℃ to RT and incubated for 60 min, then washed them 3 times. Next, the Cy3-conjugated goat anti-rabbit IgG (1:100) was added and incubated at RT for 60 min in the dark, and rinse cells 3 times. Finally, the nucleus was stained with DAPI prior to analysis by microscope. The immunofluorescence was analyzed by microscopy using an excitation wavelength at 550 nm.
